# Thermal decarboxylation for the generation of hierarchical porosity in isostructural metal–organic frameworks containing open metal sites†

**DOI:** 10.1039/d1ma00163a

**Published:** 2021-07-14

**Authors:** Hannah F. Drake, Zhifeng Xiao, Gregory S. Day, Shaik Waseem Vali, Wenmiao Chen, Qi Wang, Yutao Huang, Tian-Hao Yan, Jason E. Kuszynski, Paul A. Lindahl, Matthew R. Ryder, Hong-Cai Zhou

**Affiliations:** Neutron Scattering Division, Oak Ridge National Laboratory Oak Ridge Tennessee 37831 USA rydermr@ornl.gov; Department of Chemistry, Texas A&M University College Station Texas 77843 USA zhou@chem.tamu.edu; Department of Biochemistry and Biophysics, Texas A&M University College Station Texas 77843 USA; Department of Materials Science, Texas A&M University College Station Texas 77843 USA

## Abstract

The effect of metal-cluster redox identity on the thermal decarboxylation of a series of isostructural metal–organic frameworks (MOFs) with tetracarboxylate-based ligands and trinuclear μ_3_-oxo clusters was investigated. The PCN-250 series of MOFs can consist of various metal combinations (Fe_3_, Fe/Ni, Fe/Mn, Fe/Co, Fe/Zn, Al_3_, In_3_, and Sc_3_). The Fe-based system can undergo a thermally induced reductive decarboxylation, producing a mixed valence cluster with decarboxylated ligand fragments subsequently eliminated to form uniform mesopores. We have extended the analysis to alternative monometallic and bimetallic PCN-250 systems to observe the cluster's effect on the decarboxylation process. Our results suggest that the propensity to undergo decarboxylation is directly related to the cluster redox accessibility, with poorly reducible metals, such as Al, In, and Sc, unable to thermally reduce at the readily accessible temperatures of the Fe-containing system. In contrast, the mixed-metal variants are all reducible. We report improvements in gas adsorption behavior, significantly the uniform increase in the heat of adsorption going from the microporous to hierarchically induced decarboxylated samples. This, along with Fe oxidation state changes from ^57^Fe Mössbauer spectroscopy, suggests that reduction occurs at the clusters and is essential for mesopore formation. These results provide insight into the thermal behavior of redox-active MOFs and suggest a potential future avenue for generating mesoporosity using controlled cluster redox chemistry.

## Abstract 2

The effect of metal-cluster redox identity on the thermal decarboxylation of a series of isostructural metal–organic frameworks (MOFs) with tetracarboxylate-based ligands and trinuclear μ_3_-oxo clusters was investigated.

## Graphical abstract

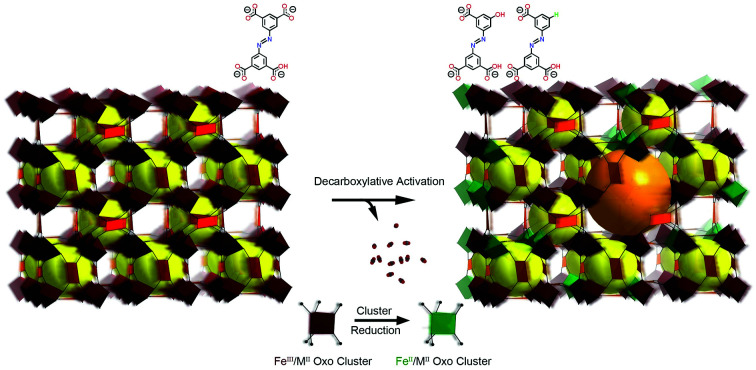

## Introduction

Porous materials such as metal–organic frameworks (MOFs) are of significant interest due to their catalytic,^1,2^ biomimetic,^3,4^ and gas storage^5–7^ applications. The wide variety of applications arise from the chemical versatility, site-specific tunability, and high crystallinity of MOF materials.^8–11^ Many MOFs show high permanent porosities. However, the pore diameters are typically in the microporous range (smaller than 2 nm), limiting them to small molecule-based applications and surface chemistry. As larger pores are ideal for applications such as heterogeneous catalysis, post-synthetic dopant modifications for gas storage, and large molecule guest delivery, the augmentation of MOF pore sizes to the mesoporous (2–50 nm) or even the macroporous (over 50 nm) scale is highly desirable.^12–15^ However, while prior research has focused on systems such as isoreticular expansion, building larger ligands with the same geometry to produce expanded versions of existing MOFs, these processes often result in either interpenetrated or unstable systems,^16^ as opposed to the desired mesoporous material. Hence, ongoing research into mesopore generation has focused on defect engineering and hierarchical porosity.^15^

The integration of multi-domain or multi-level pore sizes and environments within a single framework scaffold is often referred to as hierarchical porosity. Many approaches towards building hierarchical porosity are actively studied, such as through templated synthesis, multi-linker approaches, post-synthetic labile linker installation, and post-synthetic cluster removal.^2,12–15,17,18^ However, many of these strategies are labor-intensive, utilize expensive reagents, or require lengthy post-synthetic manipulations. The tuning of hierarchically porous structures in MOFs often involves the structural modification of the material and the enhancement of selectivity towards the desired application. For instance, hierarchical induction can offer a unique opportunity to engineer free coordination sites within a framework scaffold by removing coordinating ligands.

The importance of open coordination sites in MOFs is well established,^19^ particularly in gas adsorption.^20,21^ Open metal sites are produced either through the removal of neutral donor molecules (typically solvents) from metal coordination sites facing into the pores or through the removal of structural ligand centers, forming defect sites near the open metal coordination site.^8,10,22^ These sites typically have a stronger affinity towards common guest molecules, such as carbon dioxide, methane, and hydrogen, because of their greater degree of polarization relative to the organic backbone of the MOF.^10,23^

In a previous report,^22^ we showed that the Fe_3_ μ_3_-oxo-based redox-active material, PCN-250 (also called MIL-127),^24^ can undergo reductive decarboxylation when heated to 220 °C, producing a uniform sized mesopore and a mixed-valent iron(ii,iii) cluster. Herein, we have expanded the work to include a series of isostructural MOFs, both monometallic (Al_3_, In_3_, Fe_3_, Sc_3_) and bimetallic (Fe/Ni, Fe/Mn, Fe/Co, Fe/Zn), to confirm if these systems can also undergo the same thermal decarboxylation with resultant mesopore and open metal site formation. Our results show that the presence of redox-active metals, even in mixed-valent bimetallic systems, can undergo thermal decarboxylation, whereas redox-inactive metals cannot. To show the benefits of this system we have evaluated the ability to store carbon dioxide and methane. We found that introducing open metal sites through hierarchical porosity induction improved the gas storage performance.

## Results and discussion

**Fig. 1:**
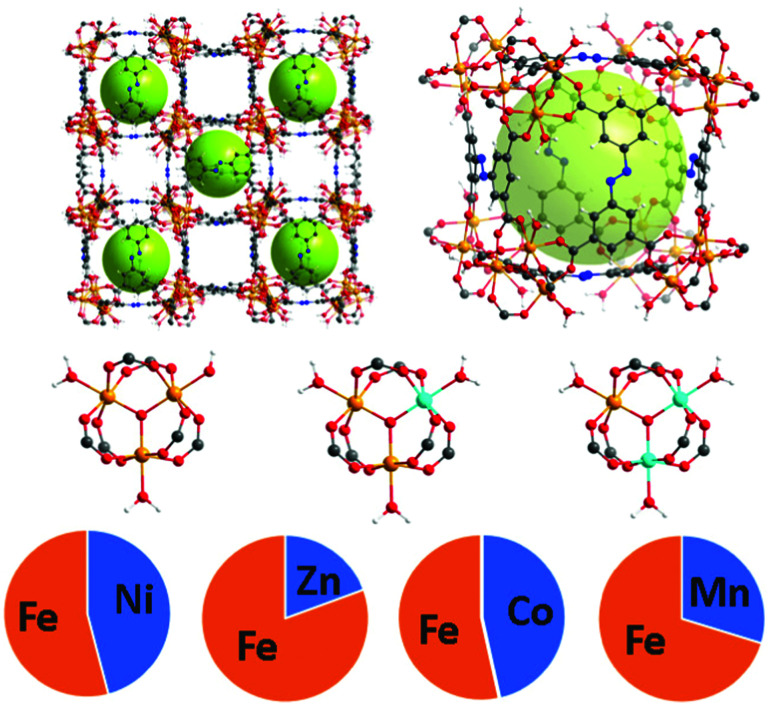
The structure of PCN-250 with mixed metal clusters Fe/M where M = Ni (45.9%), Zn (19.5%), Co (46.5%), and Mn (29.5%). The relative percentages of each metal were determined using ICP.

The PCN-250 series was investigated because the monometallic iron form has been studied thoroughly in the literature. As hierarchical porosity may involve the generation of defects in a scaffold,^25^ it is desirable to have high denticity ligands (tridentate or higher) in the parent scaffold to prevent structural collapse. The ligand used, 3,3′,5,5′-azobenzenetetracarboxylate (H_4_ABTC), is relatively cost-effective, enabling the synthesis of numerous MOF variants at reasonable yields.^5,26–28^ PCN-250 has isostructural variants of both redox-active and redox-inactive clusters generated *via* one-pot solvothermal methods in high yield.^29–32^ Both redox-active (Fe_3_, Fe/Ni, Fe/Mn, Fe/Co, and Fe/Zn) and redox-inactive metal clusters (Al_3_, In_3_, and Sc_3_) were studied to understand the influence on the hierarchical porosity generation. To control the metal ratios in the MOF, the heterogeneous metal clusters were presynthesized before synthesis of the MOF (procedure in ESI†). The ratios of the metals were maintained from preformed cluster to MOF (Fig. 1). Powder X-ray diffraction (PXRD) confirmed that the 8 MOF materials are isostructural (Fig. S2, ESI†). Additionally, pore size distribution analysis of the N_2_ adsorption results showed that each material reproduced the known microporous structure of PCN-250 (Fig. S32, S33 and S36, S37, ESI†), and the metal concentrations in the clusters were determined by inductively coupled plasma (ICP) analysis (Fig. 1).

### Thermogravimetric-mass spectrometry analysis

Open metal sites can be generated in PCN-250-Fe_3_*via* thermal decarboxylation.^22^ Long term cycling of the material at 185 °C ultimately leads to the generation of hierarchical porosity within the structure as the decarboxylation process goes to completion. This produces 37 Å mesopores in PCN-250 that result from metal-cluster removal.^22^ This process is associate with a color change, as the ordinarily red/orange powder becomes a dark brown after activation, indicating a change in the metal oxidation state. The formed mixed valent iron(ii,iii) cluster can be stabilized in an inert atmosphere, such as Ar or N_2_. Altering the material to the mixed metal variants (Fe/Ni, Fe/Mn, Fe/Co, and Fe/Zn) also resulted in an observable color change to a similar dark brown, near black color, although the same change is not observable for the Al_3_, In_3_, or Sc_3_ systems.

**Fig. 2:**
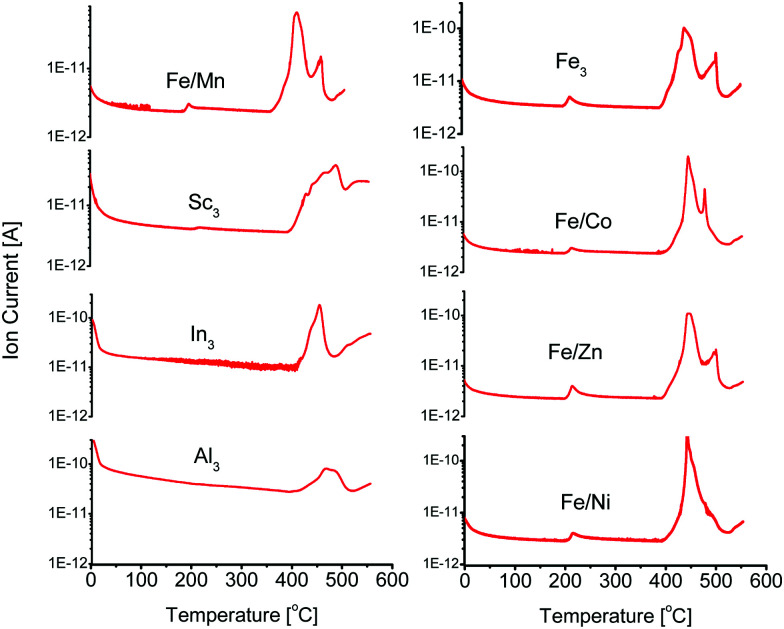
Carbon dioxide evolution through decarboxylation as seen by thermogravimetric-mass spectrometry analysis. Carbon dioxide is evolved from Fe/M where M = Fe, Co, Zn, Ni, or Mn, but is not evolved for the Sc, Al, or In isomorphs.

Additionally, TGA results of these bimetallic based MOFs show a step-like mass loss between 160 °C and 220 °C. This temperature range is comparable to the standard activation temperatures of PCN-250. The exact activation temperature and mass loss % depended on the identity of the Fe/M component (Table 1). TGA-MS analysis (Fig. S8–S23, ESI†) of these mass loss events show that CO_2_ was the main gas released at these temperatures. The CO_2_ evolution temperatures are significantly lower than what would be observed for the free ligand, with H_4_ABTC decomposing at 410 °C (Fig. S29, ESI†) with no noticeable lower temperature partial loss, suggesting that the decomposition is a synergistic effect from redox metal coordination (Fig. 2). The preformed clusters of PCN-250 (Fe_3_, Fe/Co, Fe/Mn, Fe/Ni, and Fe/Zn) were also examined by TGA-MS (Fig. S24–S28, ESI†), but the redox-inert cluster variants (Al_3_, In_3_, and Sc_3_) were not able to be synthesized according to the methods here. CO_2_ did not evolve using this method for the Al_3_, In_3_, or Sc_3_ cluster containing MOFs. TGA-MS results for these clusters did not exhibit the noticeable step-like drop in their TGA curve shape, as observed with the bimetallic MOFs. The lack of CO_2_ evolution is consistent with that previously reported for PCN-250-Al_3_.^22^ It is also consistent with what has been shown for the PCN-250 mixed metal species regarding their TGA curve shape.^29^

Comparison of decomposition and decarboxylation temperatures relative to cluster identity

**Table:** 

	Fe	Fe/Ni	Fe/Mn	Fe/Co	Fe/Zn
Cluster content (%)	100	54.1/45.9	70.2/29.8	53.4/46.6	80.5/19.5
Cluster decomp. temperature (°C)	275	285	205	195	300
MOF decarboxylation temperature (°C)	220	210	160	208	190
MOF decarboxylation mass % loss	3.2	1.8	12.6	1.7	2.4
MOF decomposition temperature (°C)	415	425	440	450	430

a Preforming Al_3_, Sc_3_, In_3_ as μ_3_-oxo clusters using the methods to make the other clusters in this study were unsuccessful. These clusters could only be generated *in situ* during MOF formation.

**Table:** 

	Al	In	Sc
MOF decomposition temperature (°C)^a^	400	370	455

### Mössbauer spectroscopy

**Fig. 3:**
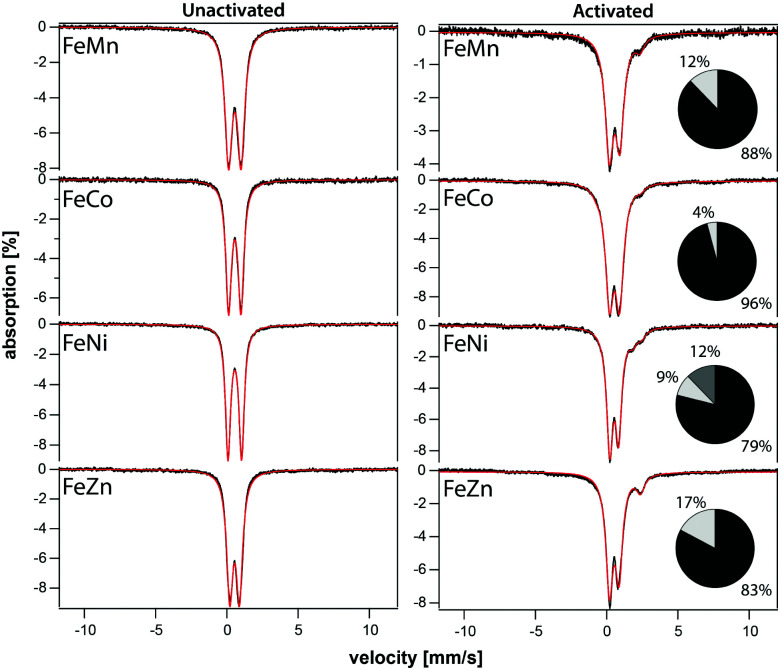
Mössbauer spectra of unactivated (left) and activated (right) mixed-metal MOFs. Spectra were collected at 5 K and 0.05 T with the applied field parallel to the gamma radiation. Black hashmarks are data; red lines are simulations. Black column inset quantifies the dominant quadrupole doublet and any minor amount of magnetic material extending from the baseline. The light grey columns indicate the percentage of Fe^II^ sites.

The iron component of four mixed-metal PCN-250 MOFs was investigated using ^57^Fe Mössbauer spectroscopy, which is sensitive to the oxidation state and ligand environment of ^57^Fe nuclei. Although non-iron components of the material are Mössbauer-silent, they can still indirectly influence the spectral properties. Spectra of the four unactivated samples at low temperature (5 K) and low-field (0.05 T) were dominated (>95% spectral intensity) by a quadrupole doublet (Fig. 3) with isomer shift *δ* = 0.54 ± 0.02 mm s^−1^ and quadrupole splitting Δ*E*_Q_ ranging from 0.67 to 0.95 mm s^−1^. These parameters suggest that the iron in the unactivated mixed-metal MOFs is primarily Fe^III^. The ‘all-iron’ PCN-250 material investigated previously had identical *δ* and similar Δ*E*_Q_, namely 0.61 mm s^−1^.^22^ Linewidths *Γ*, which reflect the heterogeneity of the iron environment within the MOF, varied from 0.44 mm s^−1^ (for FeNi) to 0.61 mm s^−1^ (FeMn). For the all-iron MOF, *Γ* was 0.48 mm s^−1^.^22^ Activation of the mixed-metal MOFs at 185 °C under vacuum resulted in significant spectral changes. For the major quadrupole doublet, Δ*E*_Q_ declined most dramatically for FeNi and least for FeZn, whereas *δ* was uniformly unchanged and *Γ* increased an average of 20%. These changes indicate that activation had no effect on the oxidation state of iron associated with the doublet, but the environment around the iron was affected, including increased heterogeneity. Between 4% and 21% of the iron in these MOFs were dramatically affected by activation, specifically reduced to the Fe^II^ state. The Fe^II^ site in the FeZn and FeMn MOFs was similar, with *δ* = 1.26 mm s^−1^ and Δ*E*_Q_ = 2.17 mm s^−1^. The FeCo site had *δ* = 0.83 mm s^−1^ and Δ*E*_Q_ = 3.09 mm s^−1^. The activated FeNi MOF had two Fe^II^ sites, with *δ* = 0.99 and 1.41 mm s^−1^, and Δ*E*_Q_ = 1.7 and 2.1 mm s^−1^ (all parameters ±0.02 mm s^−1^). The Fe^II^ site in the all-iron MOF had similar parameters, namely *δ* = 1.24 mm s^−1^ and Δ*E*_Q_ = 2.3 mm s^−1^. Mössbauer spectra were collected on a model MS4 WRC (SEE Co) spectrometer. Spectra were calibrated against α-Fe foil at RT and simulated using WMOSS software.

**Fig. 4:**
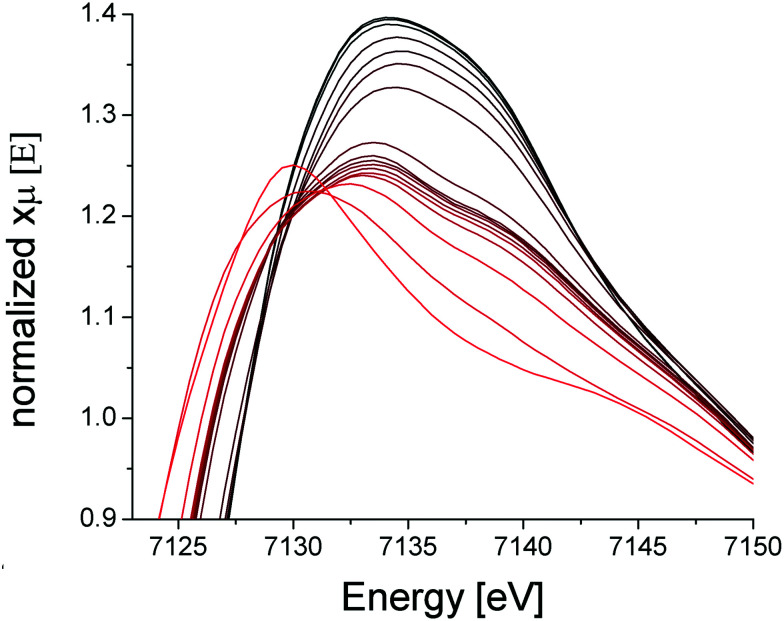
XAS spectra of PCN-250 (Fe_3_) in the range of 23 °C (black) to 450 °C (red).

X-Ray absorption spectroscopy (XAS) was collected on the PCN-250 (Fe_3_) sample during activation to further the findings of metal electronic environmental changes found in the Mössbauer spectroscopy studies. In the XAS spectra, there is a dominant white line energy peak near 7125 eV for the as-prepared sample of PCN-250 (Fe_3_). This peak corresponds to the electronic environment of coordinating ligands through a Fe–O bond to the iron center. With the increase of temperature, the white line peak of iron in PCN-250 (Fe_3_) gradually reduces its energy intensity with a relatively fast drop between 150 °C to 185 °C (Fig. 4). This energy shift indicates detachment (or dissociation) of some ligands from the metal cluster but does not indicate complete decomposition.

**Fig. 5:**
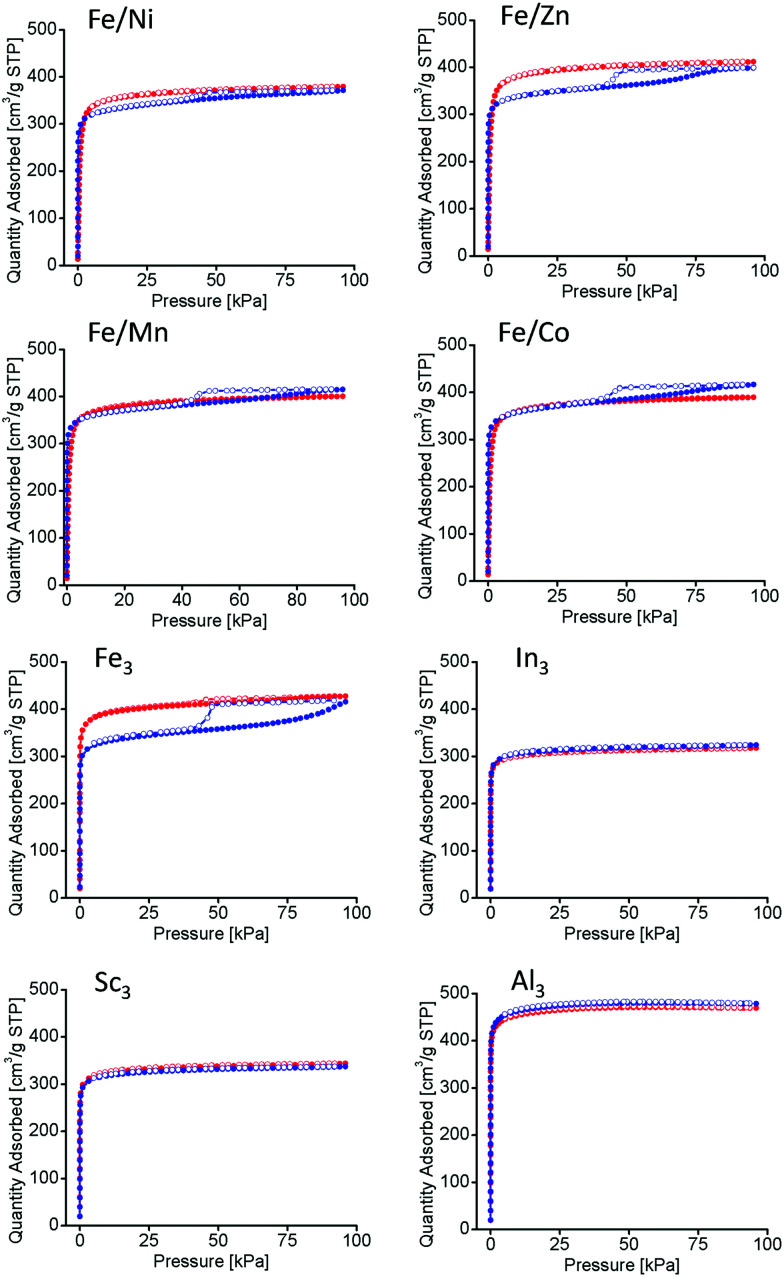
Nitrogen Isotherms at 77 K before (red) and after (blue) 100 hour thermal activation. In the Fe/Ni, Fe/Zn, Fe/Mn, Fe/Co, Fe_3_, clusters a hysteresis loop is observable indicating mesopore formation. Samples for In_3_, Sc_3_, and Al_3_ do not have mesopore formation.

Long-term thermal studies were conducted on all PCN-250 variants to observe the gradual changes in the structures related to the potential thermal decarboxylation event. Long-term activation was performed under vacuum at 185 °C for 100 hours, simulating the conditions used for BET isothermal analysis (185 °C under vacuum for 12 hours).^22^ The N_2_ isotherms at 77 K of the materials before and after long term thermal activation were compared. The monometallic iron (Fe_3_) and the bimetallic (Fe/Ni, Fe/Mn, Fe/Co, and Fe/Zn) systems exhibited a noticeable hysteresis loop, indicative of mesopore generation (Fig. 5).^33^ However, no hysteresis loop was present for the redox-inactive clusters (Al_3_, In_3_, and Sc_3_). The BET surface areas of the MOFs as determined by nitrogen adsorption methods may be seen in Table 2. As expected, the surface area is shown to decrease in the mesoporous samples.

**Table:** BET surface area, methane adsorption at 298 K, and carbon dioxide adsorption at 298 K for each of the PCN-250 isomorphs

PCN-250 structure	BET surface area (m^2^ g^−1^)	298 K CO_2_ adsorption at 110 kPa (cm^3^ g^−1^ STP)	298 K CH_4_ adsorption at 110 kPa (cm^3^ g^−1^ STP)
Fe_3_ microporous	1.628	74.06	21.26
Fe_3_ mesoporous	1.421	101.53	21.82
Fe/Ni microporous	1.514	72.14	18.11
Fe/Ni mesoporous	1.320	91.94	20.56
Fe/Co microporous	1.679	83.42	21.25
Fe/Co mesoporous	1.433	120.75	27.13
Fe/Mn microporous	1.618	82.63	18.03
Fe/Mn mesoporous	1.457	83.23	22.15
Fe/Zn microporous	1.545	74.13	20.12
Fe/Zn mesoporous	1.359	80.96	21.35
Al_3_ microporous	1.880	105.86	33.92
In_3_ microporous	1.218	68.18	17.07
Sc_3_ microporous	1.316	82.76	19.27

The post-long-term thermal activation samples were again measured by TGA-MS (Fig. S8–S23, ESI†). The results indicated the continued presence of CO_2_ evolution in the redox-active clusters. This data suggests that, although the 100 hours 185 °C activation generates significant mesoporosity levels in the redox-active systems, it does not reach a complete reduction in the MOF systems suggesting a slow, gradual process.

Our previous work showed the decarboxylated versions of H_4_ABTC *via* GC-MS after the sample underwent long-term thermal processing. To observe these ligand fragments in the isostructural MOF samples, small portions of the post-long term activation samples were decomposed in pH = 12 solution.^22^ The supernatant was tested *via* GC-MS. Small amounts of decarboxylated ligands were present in the redox active cluster samples containing MOFs but not in the redox inert samples. As reported previously, the concentration of the decarboxylation products was not observable *via*^1^H or ^13^C NMR, but was qualitatively detectable in GC-MS (Fig. S59–S69, ESI†).^22^

### Methane and carbon dioxide gas sorption

**Fig. 6:**
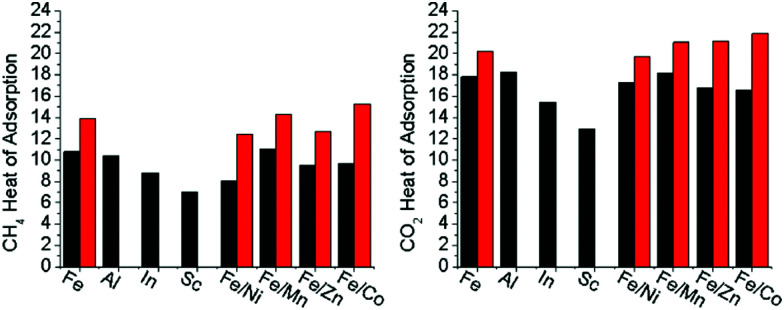
The average heat of adsorption for CH_4_ and CO_2_ for the microporous (black) and mesoporous (red) sample isomorphs. Heat of adsorption was calculated based on the experimental adsorption isotherms at 195 K, 273 K, and 298 K for each gas.

To quantify the effect of open metal site generation *via* thermal methods in the mixed metal, redox-active variants, the heat of adsorptions were calculated for both CO_2_ and CH_4_ (Fig. S51–S58, ESI†). A discussion on how Heat of Adsorption is calculated may be found in the ESI.† As both CO_2_ and CH_4_ are sensitive towards open metal sites,^10,23,34,35^ they were used to detect open metal sites being thermally generated in the hierarchically porous material compared to the base material counterparts. The heat of adsorptions were determined through isothermal analysis of the gases at 195 K, 273 K, and 298 K. The room temperature adsorption at 110 kPa of both carbon dioxide and methane are reported in Table 2. In all cases, the adsorption of each gas at room temperature increased in the presence of hierarchal porosity. In the redox-active-cluster-containing MOFs, the heat of adsorption changed for both gases, showing a dramatic increase in kJ mol^−1^ for these materials (Fig. 6). These results suggest that gas adsorption interactions increase with thermal decarboxylation-induced hierarchical porosity. This analysis correlates with TGA-MS and Mössbauer spectroscopy results, with the open reduction events, and with decarboxylation resulting in the formation of open metal sites and their resultant high gas affinities. Our results suggest the intriguing possibility of precisely tuning the heat of adsorption towards the desired application for both CO_2_ and CH_4_*via* open metal site generation utilizing this hierarchical porosity generation strategy.

### Structural activation *via* decarboxylation

**Fig. 7:**
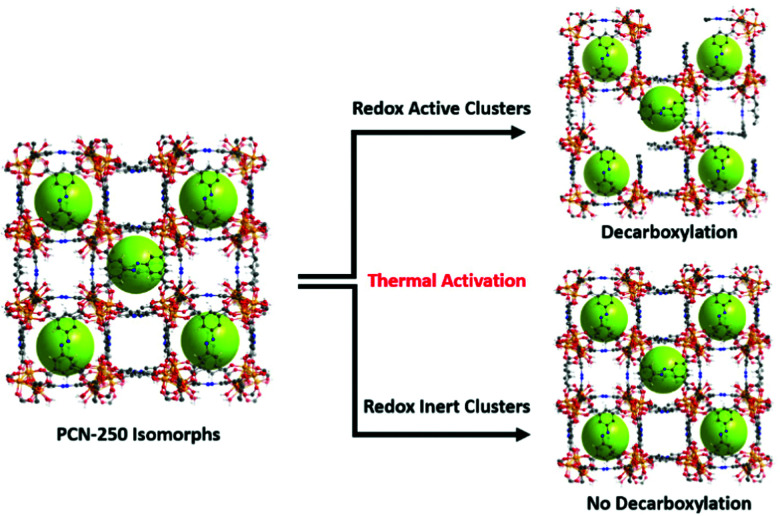
The findings of the study indicate the dependence of thermally induced decarboxylation on the redox activity of the clusters in the isomorphs of PCN-250.

The combination of the metal oxidation state change upon activation, presence of carbon dioxide generation upon reaching activation temperatures, generation of a growing hysteresis loop in nitrogen adsorption isotherms, presence of decarboxylation products in GC-MS, and the increase in the heat of adsorption towards gas guest molecules indicates that the previously reported mechanism of activation by decarboxylation for the Fe_3_ μ_3_-oxo cluster is conserved for the heterogeneous cluster containing MOFs (Fe/Ni, Fe/Mn, Fe/Zn, and Fe/Co). These clusters are all redox-active species and able to generate open metal sites. By contrast, the lack of these features in the Sc, In, and Al μ_3_-oxo cluster-based MOF data indicates that the thermal decarboxylation activation event may depend on the redox nature of the clusters. Thus, the mechanism for thermal decarboxylation may depend on the redox activity of the MOF cluster. By contrast, the non-redox active clusters in this study appear structurally stable to the thermal activation conditions used here. These insights may lead to future avenues of research where applications demanding structural thermal stability is imperative (Fig. 7).

It is perhaps of interest to note here the recent advances in redox-inert-cluster-based MOFs in various areas of research, particularly Al-MOFs.^36–38^ The findings of this work can better understand the origins of stability for these structures over their isostructural variants with redox-active clusters.

## Conclusions

This work utilized thermal decarboxylation for hierarchical porosity generation in a series of isostructural MOFs. The nature of the decarboxylation influence was investigated by using both redox-active and redox-inert cluster options for the MOF PCN-250. Upon activation at elevated temperatures for extended periods, the redox-active clusters underwent open metal site generation through decarboxylation, forming hierarchical porous structures. The decarboxylation pathway did not result in complete structural collapse but instead generated a 37 Å mesopore within the structure. The thermally generated hierarchically porous structures improved uptake performance for both carbon dioxide and methane; both showed increases in heat of adsorption upon generating the hierarchical porous materials. The thermal decarboxylation pathway offers an efficient and easy pathway for generating hierarchical porosity without using templates, multiple ligands, or extensive ligand exchanges; the process is solvent-free. These advantages and its relative ease of use and variety of starting structures render this process of potential interest for generating novel hierarchically porous materials in the future. These results also provide an improved understanding of the potential origins of thermal stability for redox-inert isostructural MOFs.

## Conflicts of interest

There are no conflicts to declare.

## Supplementary Material

MA-002-D1MA00163A-s001
