# Supplementary material for: Thermal decarboxylation for the generation of hierarchical porosity in isostructural metal–organic frameworks containing open metal sites
Source: Mater Adv. 2021 Jul 14;2(16):5487–93. doi: 10.1039/d1ma00163a (PMC8366390; doi:10.1039/d1ma00163a)
Supplement: MA-002-D1MA00163A-s001 [file MA-002-D1MA00163A-s001.pdf]

Electronic Supplementary Material (ESI) for Materials Advances.
This journal is © The Royal Society of Chemistry 2021

Supplemental Information

Thermal Decarboxylation for the Generation of Hierarchical Porosity in Isostructural Metal-Organic Frameworks Containing Open Metal Sites

Hannah F. Drake,[a,b] Zhifeng Xiao,[b] Gregory S. Day,[a,b] Shaik Waseem Vali,[c] Wenmiao Chen,[b] Qi Wang,[b] Yutao Huang,[b] Tian-Hao Yan,[b] Jason E. Kuszynski,[b] Paul A. Lindahl,[c] Matthew R. Ryder,[a,*] and Hong-Cai Zhou[b,d,†]

*rydermr@ornl.gov
†zhou@chem.tamu.edu

Table Of Contents

Experimental Procedures……………………………………………………………………………3-5

Instrumentation……………………………………………………………………………………………5

Discussion of Heat of Adsorption Calculations……………………………………………6-7

Experimental Data

^{1}HNMR (Figure S1) ………………………………………………………………………………………8

Powder X-Ray Diffraction (Figure S2-3) ………………………………………………………………9

Scanning Electron Microscopy Images (Figure S 4-7) ………………………………10-11

Thermogravimetric Analysis-Mass Spectrometry (Figure S8-30) …………………12-23

X-Ray Absorption Spectroscopy (Figure S31) ……………………………………………………23

N_2 Gas Adsorption (Figure S32-35) ……………………………………………………………24-25

Mesopore Distribution (Figure S36-37) ………………………………………………………………26

CO_2 and CH_4 Gas Adsorption (Figure (S 38-50)………………………………………27-31

Heat of Adsorption (S50-58) ……………………………………………………………………31-33

Gas Chromatography-Mass Spectrometry (S59-68)……………………………………34-38

Infrared Spectroscopy (S69-70)……………………………………………………………………………39

References ……………………………………………………………………………………………………40

Experimental Procedures

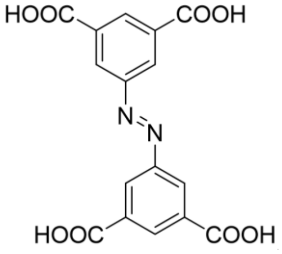

Synthesis of ABTC:3,3',5,5' Azobenzene-tetra-carboxylic acid (H_4ABTC):

The synthesis of H_4ABTC followed previously reported procedures to synthesize this ligand with slight modification.[1-4] To a 100 mL flask was added 5-nitroisophthalic acid (2 g, 9.4 mmol), sodium hydroxide (6.5 g, 160 mmol), and DI water (25 mL). To a separate 100 ml flask was added D-glucose (11.5 g, 64 mmol) and DI water (25 mL). Both flasks were heated to 70 °C and stirred until both solutions were clear. The solutions were combined and stirred for 12 hours at 70 °C under bubbling air into the solution. Note: bubbling must be gentle. The dark brown solution was cooled on an ice bath. The yellow-brown solids could be collected by centrifugation. The solids were dissolved in excess water and acidified with conc. Hydrochloric acid in an ice bath. The product was isolated by centrifugation, washed once with acetone, and dried in air. Yield: 83%.

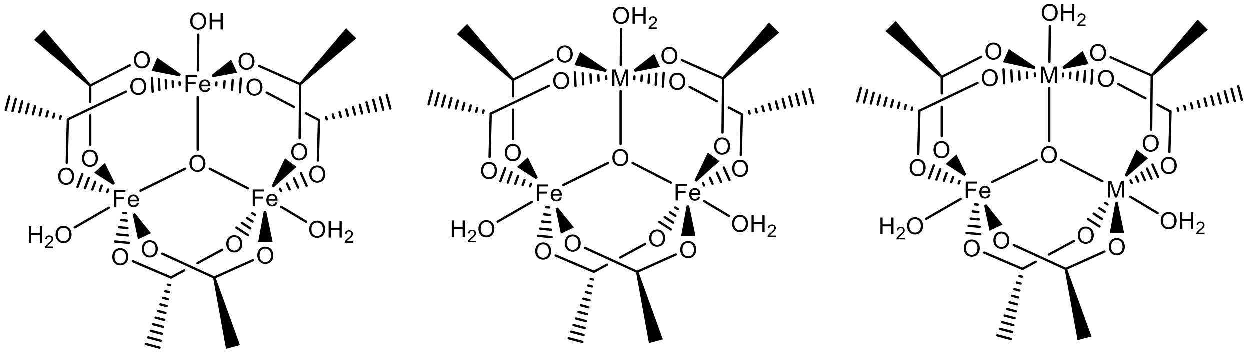

Synthesis of PCN-250 clusters:

The mixed metal clusters were synthesized according to previously reported procedures with some slight modifications.[2, 5] To a 250 mL beaker was added sodium acetate (21.0580 g, 256.70 mmol) and water (20 mL). This was dissolved by sonication. To a second 250ml beaker was added $Fe(NO_3)_3$ x $9H_2O$ (4.0701 g, 16.83 mmol) and $M(NO_3)_2$ x X H_2O salt (M = Co, Ni, Zn, or Mn, 51.89 mmol). This was dissolved in water (20 mL) via sonication. The two beakers were combined and stirred at room temperature for 12 hours. The precipitate was filtered in water with minimal washings before being air-dried to yield the pure cluster product. The preformed clusters utilized in this study were Fe_3, Fe/Zn, Fe/Mn, Fe/Ni, and Fe/Co. Other combinations were attempted, including In_3, Al_3, Sc_3, and (Al, In, Sc)/(Fe, Mn, Ni, Co, Zn) cluster pre-synthesis, but no other cluster types can be performed successfully as determined by metal content by ICP and ^{1}HNMR.

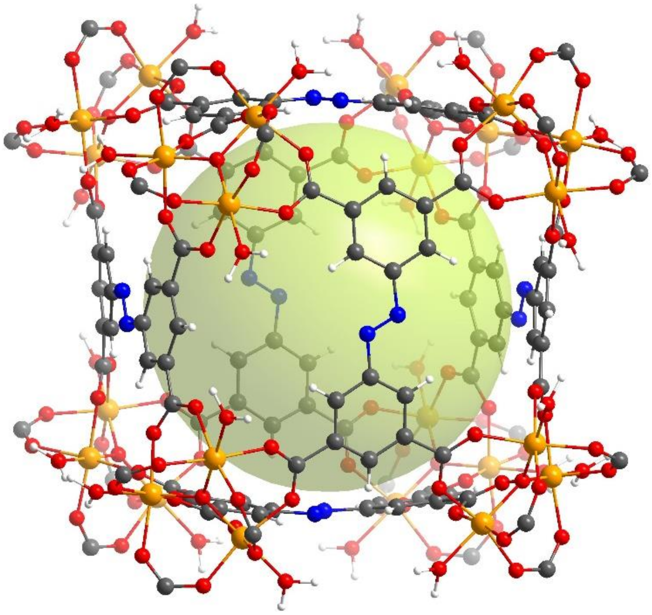

Synthesis of PCN-250 MOF:

To a 20 mL pyrex vial was added H_4ABTC (180 mg) and DMF (10 mL). To a second Pyrex vial was added a metal nitrate salt or preformed cluster (540 mg), DMF (2 mL), and glacial Acetic Acid (6mL). The two vials were sonicated until the contents had dissolved. After dissolution, the contents of the vials were combined, filtered, and immediately placed into a 150 °C oven. The MOFs were collected after 48 hours. The products were washed 3x with fresh DMF and allowed to sit in fresh DMF for 24 hours after washing. The samples were then transferred into a soxhlet extractor for two days with Methanol extraction to yield the pure microporous MOF. The samples were dried in air before analysis. Yield: 53%

Generation of hierarchical porosity through decarboxylation:

Generation of hierarchically porous samples occurred through two methods. In the first method, small quantities of the samples, up to 100 mg, were loaded in a BET tube and were heated on the activation port of Micromeritics ASAP 2420 for 12 hours at 185 °C. In order to generate a significant quantity of mesopores in these structures for a larger scale sample (up to 1 g), continual thermal activation under vacuum at 185 °C was utilized on a Schlenk line for 100 hours.

Analysis by Mossbauer sample preparations:

The Iron-containing samples were analyzed via ^{57}Fe Mössbauer spectroscopy. The thermally activated samples were activated under vacuum and heat on a schlenk line. To ensure no contamination from air, all samples were sealed on the schlenk line and transferred to a glovebox, and sealed in e-icosane for Mossbauer sample preparation. Samples were then transferred immediately into a liquid nitrogen bath for storage after removal from the inert atmosphere once the E-icosane shielding the sample had properly set.

Analysis by LC-MS sample preparations:

Small samples of the thermally treated MOFs were decomposed via 1 mL ammonium hydroxide solution (250ul ammonium hydroxide in 1ml water) at 85 °C for 12 hours. The orange solutions were then filtered

and analyzed by LC-MS after dilution with water. Note: ABTC as a free ligand is highly insoluble in most solvents. In order to maintain solubility, the ligand must be in a basic aqueous solution for analysis.

Analysis by Inductively Coupled Plasma Mass Spectrometry (ICP-MS) sample preparation:

Conc. HNO_3 (500 µL) was used to dissolve the sample in a vial at 80 °C overnight. A dilution of this was made by first taking 200 µL of this solution and diluting it to 10ml. Then a second dilution was made, taking 100µL from this solution and diluting to 10ml.

Instrumentation

Powder X-ray diffraction (PXRD) was carried out with a Bruker D8-Focus Bragg-Brentano X-ray Powder Diffractometer equipped with a Cu sealed tube (λ = 1.54178 Å) at 40 kV and 40 mA.

Scanning Electron Microscopy (SEM) measurements were carried out on JEOL JSM-7500F. JEOL JSM-7500F is an ultra-high-resolution field emission scanning electron microscope (FE-SEM) equipped with a high brightness conical FE gun and a low aberration conical objective lens.

Thermal Gravimetric Analysis-Mass Spectrometry (TGA-MS) was performed using a Mettler-Toledo TGA/DSC STAR*e*-1 system which was equipped with a GC100 gas controller. The MS data were collected using a ThermoStar Gas Analyzer. The system was sealed from the outside environment during analysis and collected with the carrier gas Ar.

N_2, CO_2, and CH_4 sorption measurements were conducted using a Micromeritics ASAP 2020 and 2420 system. The thermal activation before analysis profiles was 185 °C at 10 hours under vacuum for all samples.

Gas Chromatography-Mass Spectrometry (GC-MS) was performed using a Thermo Scientific DSQ II GCMS. An ESI analysis was used.

Inductively Coupled Plasma Mass Spectrometry (ICP-MS) -Calibration standards were prepared from certified reference standards from RICCA Chemical Company. Samples were further analyzed with a PerkinElmer NexION 300D ICP mass spectrometer. The resulting calibration curves have a minimum R2 = 0.9999. Additionally, to maintain accuracy, quality control samples from certified reference standards and internal standards were utilized. The individual results of the triplicate samples were averaged to determine the metal concentration.

^{57}Fe Mössbauer measurements: Samples were transferred into a cup designed to fit the Mössbauer Spectrometer under an inert atmosphere and were rapidly frozen in liquid N_2 in an anaerobic refrigerated glove box. Mössbauer data was then collected at 5K and 150K on an MS4 WRC or LHe6T spectrometer (SEE Co., Edina, MN). The data was analyzed and simulated using WMOSS software (http://www.wmoss.org/). The instrument was calibrated at room temperature with α-iron foil.

For X-ray absorption spectroscopy (XAS), the sample was packed inside the sample holder under a constant flow of high purity helium. Helium flow was used during thermal studies as well. The XAS studies were conducted at the Advanced Photon Source at Argonne National Laboratory under the direction of Dr. Di-Jia Liu.

Discussion of Heat of Adsorption (HOA) Calculations (Figures S43-S50)

Heat of adsorptions for both methane and carbon dioxide were calculated based upon the Langmuir[8-10] fits of the isotherm data at 195 K, 273 K, and 298 K. Langmuir fits for the three data sets were conducted using the OriginPro 8.5 software by taking the original isotherm data points and utilizing the non-linear curve fit to the single-site Langmuir equation:

$$Q = \frac{q_{sat}(K * P^n)}{1 + K * P^n}$$

P, the pressure (kPa) was set to be the independent variable in the fit, while Q, the quantity adsorbed (cm^3/g STP), was set as the dependent variable. The three parameters, q_{sat}, K, and n, were varied as part of the fit.

An example of the resulting parameters for the six isotherms and their corresponding error values are shown in the table below:

Table HOA1: Parameter set for PCN-250-Fe/Zn mesoprorous

Isotherm	q_{sat}	K	n	R^2	Chi^2
CH_4 195 K	200.82171 (0.67129)	0.02032 (1.46514*10^{-4})	1.03153 (0.00357)	0.99997	0.0423
CH_4 273 K	165.0629 (0.82983)	0.00234 (7.40186 *10^{-6})	0.99768 (6.83626*10^{-4})	0.99999	5.0693*10^{-5}
CH_4 298 K	200.82477(3.33589)	0.00115(1.54962*10^{-5})	0.99143(0.00109)	0.99999	5.2043*10^{-5}
CO_2 195 K	311.46515(2.3145)	0.31957 (0.01183)	0.93866 (0.00284)	0.99944	0.0909
CO_2 273 K	308.40838(8.57426)	0.0061 (8.09398 *10^{-5})	1.02259 (0.0107)	0.99981	0.2461
CO_2 298 K	465.60928 (14.78156)	0.00254 (5.6267 *10^{-5})	0.94409 (0.00356)	0.99998	0.0101

Each of these equations was then used to calculate adsorption values over a wider pressure range as compared to the experimental data in Microsoft Excel([=((B5*B6*L1^(B7))/(1+B6*L1^(B7)))] where values for q, b, and v are entered into B5, B6, and B7 respectively, and pressure ranges are entered into column L). The calculated data points for each gas at the three temperatures were then compared to find pressure values with matching adsorption quantities through the use of Excel's Match function ([MATCH(N210,R:R,0)] as an example. In this, the match function within Excel is finding the location within the row from the 273 K data (Column R) where the value in the row (the quantity adsorbed) matches the quantity adsorbed value in the given row the 298 K data (N210))). For this data, each isotherm was varied only by 0.1 mbar, providing a high degree of data overlap for matching.

In one column, the Match function was utilized to find the quantity adsorbed data row from the 298 K isotherm that matched a given row for the 195 K isotherm quantity adsorbed. In another column, the Match function was utilized in the same manner to match the 298 K and 273 K isotherms.

Following this, a combined IFNA and IF function was utilized to find the location where both 195 K and 273 K quantity adsorbed points matched to a 298 K point.

[=IFNA(IF((AND(Y210>1,Z210>1)),ROW()),FALSE)].

The IF function looks to see if there is a value in row Y (corresponding to 273 K rows with quantities adsorbed matching a 298 K value) and row Z (corresponding the comparable 195 K data) that are greater than 1, if there are, then it gives the row number that corresponds to that location. If both of those things are not true, then the IFNA function responds with a result of "FALSE."

We can use the SMALL function on our IFNA row to find the nth smallest number in the row from this info. We utilize a value range column (=SMALL(AA:AA,A12)to allow us to iterate the small function to get the next smallest value in each subsequent row.

Once we have the row numbers, which correspond directly to quantity adsorbed values for 298 K, we use an INDEX function to search through the entire file and find the data that is in the given row number. In this INDEX function, the $1:$1048576 tells the function that it is looking through the entire sheet, the AB column is a list of row numbers, so AB2 is our first-row number of interest, 14 refers to column N. Together, these two give us cell N210, the value shown, 2.51, is the value given in cell N210. This is the lowest quantity adsorbed value that matches between the three isotherms.

Additional INDEX functions are then used to find the corresponding pressure values for the given quantity adsorbed.

The pressures are located in Column 12 (L) and row 210 (shown in AB2).

Once all three pressure values and their corresponding quantities adsorbed were collected, they were analyzed using the Clausius-Clapeyron[11-13] equation in the form:

$$lnP = \frac{Q}{R} * \frac{1}{T}$$

The natural log of the pressure values was taken and compared against 1/T (1/298, 1/273, and 1/195). This gave us a series of lines with three data points each. We calculated the slope of each line using Excel's SLOPE function, giving us the value for Q/R. We then multiplied this by R (0.008314 kJ/mol*K to give us the value of the heat of adsorption for each quantity adsorbed. We were then able to graph Heat of adsorption (in kJ/mol) versus the quantity adsorbed (in cm^3/g STP). As shown in Figures S43-S50.

Experimental Data

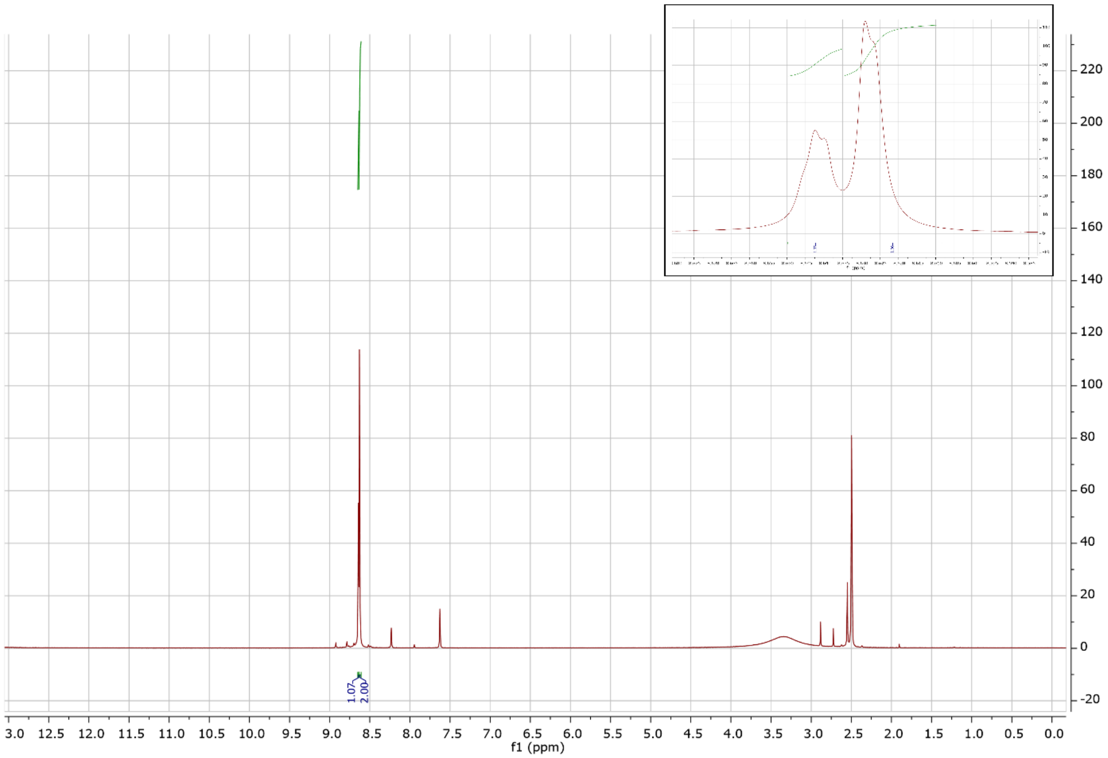

Figure S1. ^{1}HNMR of H_4ABTC in D_6-DMSO. Integrations are shown in green indicating a 1:2 ratio expected.

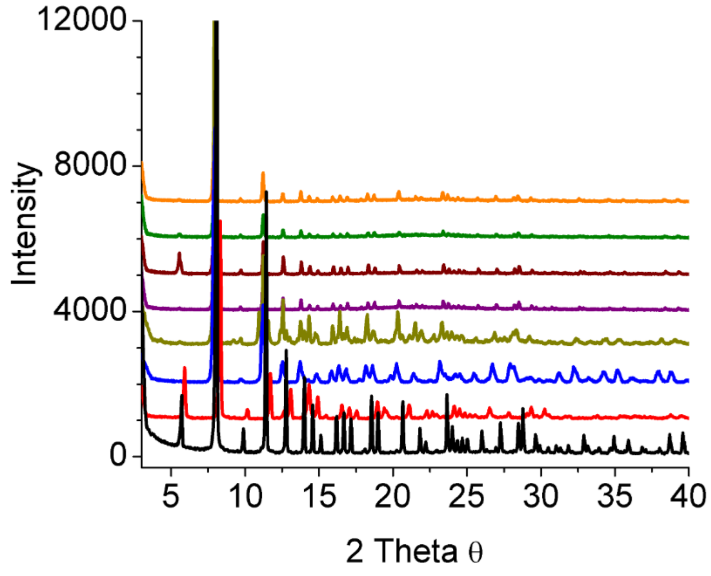

Figure S2. A comparison of experimental PXRD patterns of the isomorphs of PCN-250 before thermal activation. The crystallinity of the experimental patterns is observed to be maintained for all isomorphs. Fe_3 MOF (black), Al_3 MOF (red), In_3 MOF (blue), Sc_3 MOF (gold), Fe/Ni MOF (purple), Fe/Zn MOF (maroon), Fe/Co MOF (green), Fe/Mn (orange).

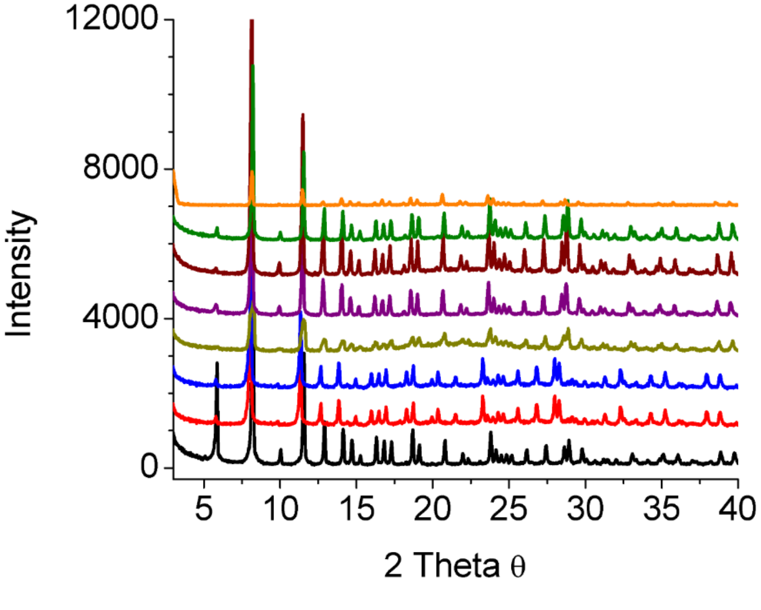

Figure S3. A comparison of experimental PXRD patterns of the isomorphs of PCN-250 after heat cycling. The crystallinity of the experimental patterns is observed to be maintained for all isomorphs. Fe_3 MOF (black), Al_3 MOF (red), In_3 MOF (blue), Sc_3 MOF (gold), Fe/Ni MOF (purple), Fe/Zn MOF (maroon), Fe/Co MOF (green), Fe/Mn (orange).

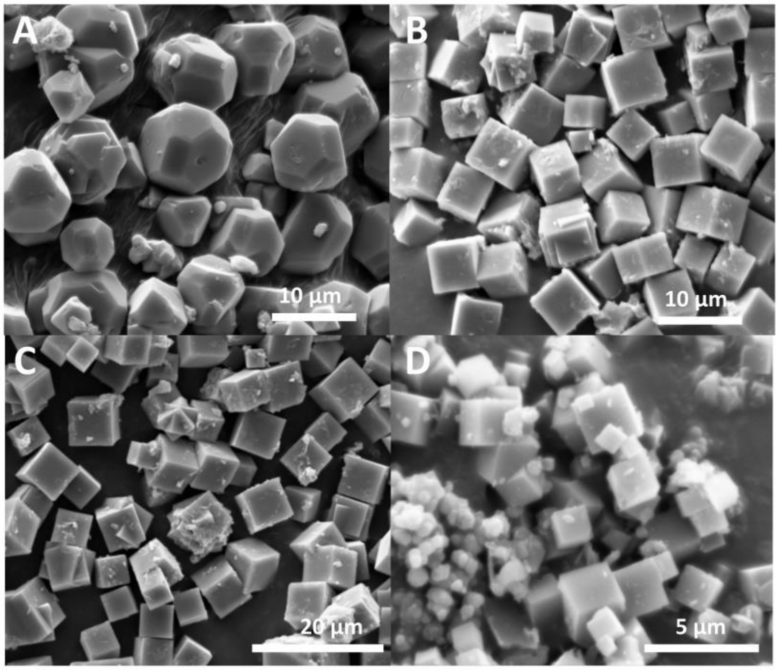

Figure S4. SEM images of A) Fe_3 MOF, B) Sc_3 MOF, C) In_3 MOF, and D) Al_3 MOF before thermal treatment.

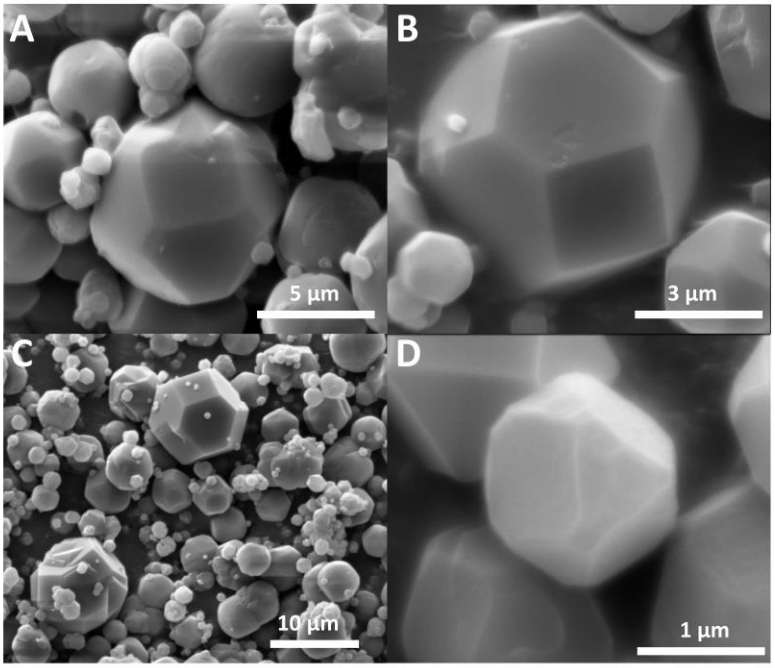

Figure S5. SEM images of A) Fe/Ni MOF, B) Fe/Mn MOF, C) Fe/Zn MOF, and D) Fe/Co MOF before thermal treatment.

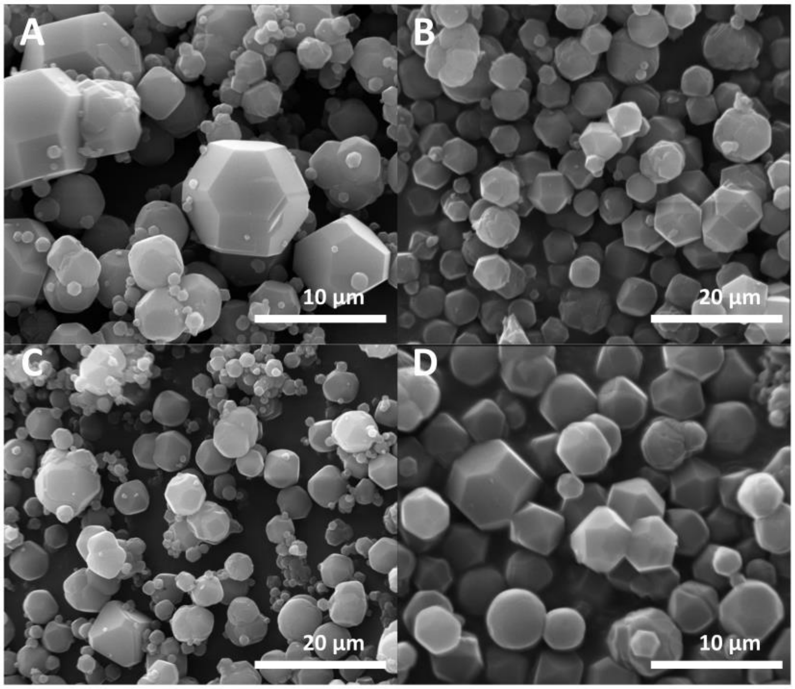

Figure S6. SEM images of A) Fe/Ni MOF, B) Fe/Mn MOF, C) Fe/Zn MOF, and D) Fe/Co MOF after thermal treatment.

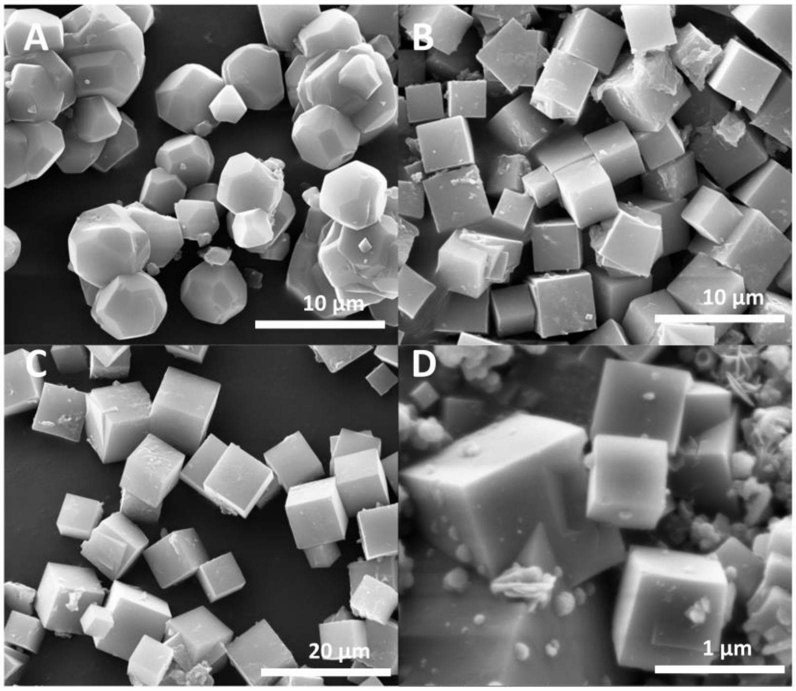

Figure S7. SEM images of A) Fe_3 MOF, B) Sc_3 MOF, C) In_3 MOF, and D) Al_3 MOF after thermal treatment.

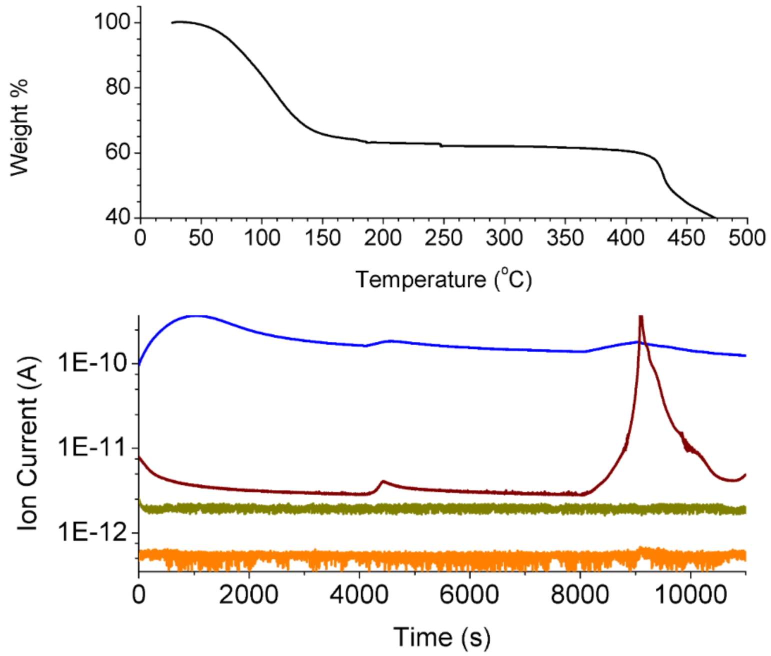

Figure S8. TGA curve(black) of the Fe/Ni MOF before thermal cycling where specific gases, H_2O (18amu blue), MeOH (31 amu gold), CO_2(44amu maroon), and DMF (73 amu orange), were monitored for the temperature of gas release from the sample.

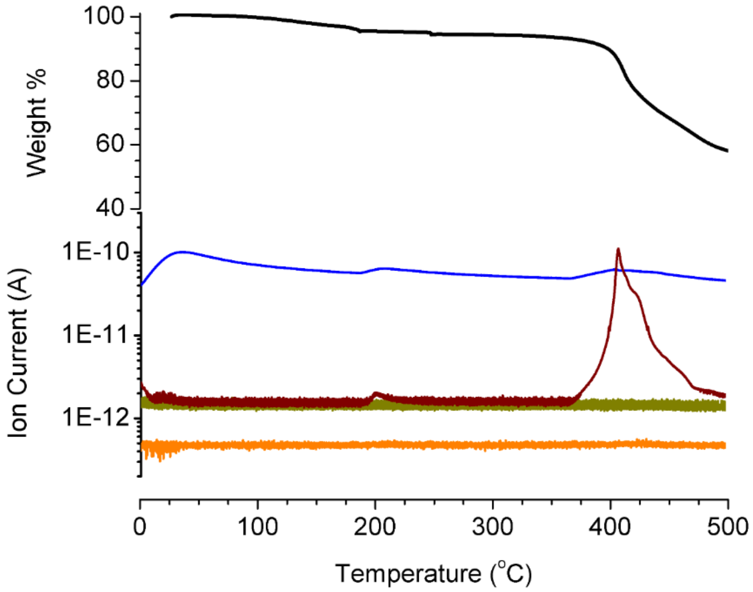

Figure S9. TGA curve(black) of the Fe/Ni MOF after thermal cycling where specific gases, H_2O (18amu blue), MeOH (31 amu gold), CO_2(44amu maroon), and DMF (73 amu orange), were monitored for the temperature of gas release from the sample.

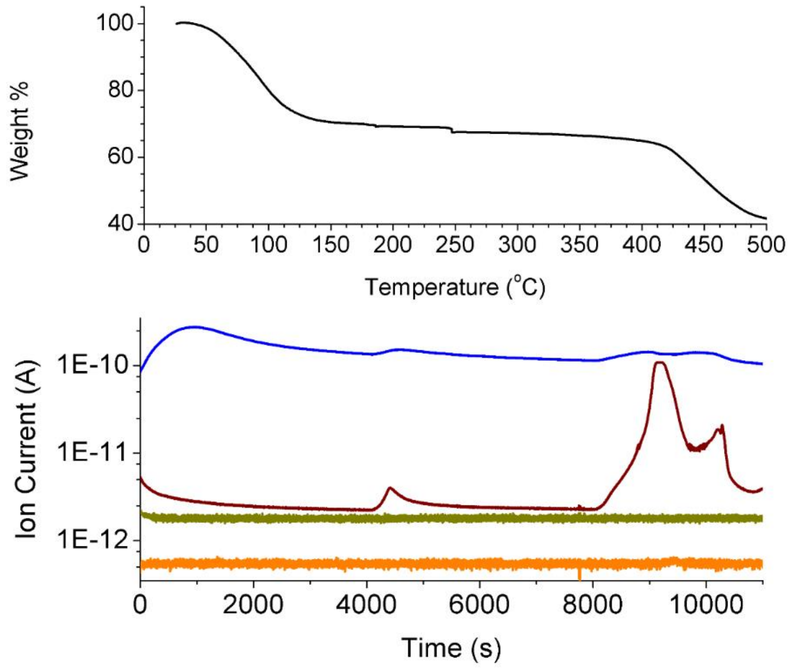

Figure S10. TGA curve(black) of the Fe/Zn MOF before thermal cycling where specific gases, H_2O (18amu blue), MeOH (31 amu gold), CO_2(44amu maroon), and DMF (73 amu orange), were monitored for the temperature of gas release from the sample.

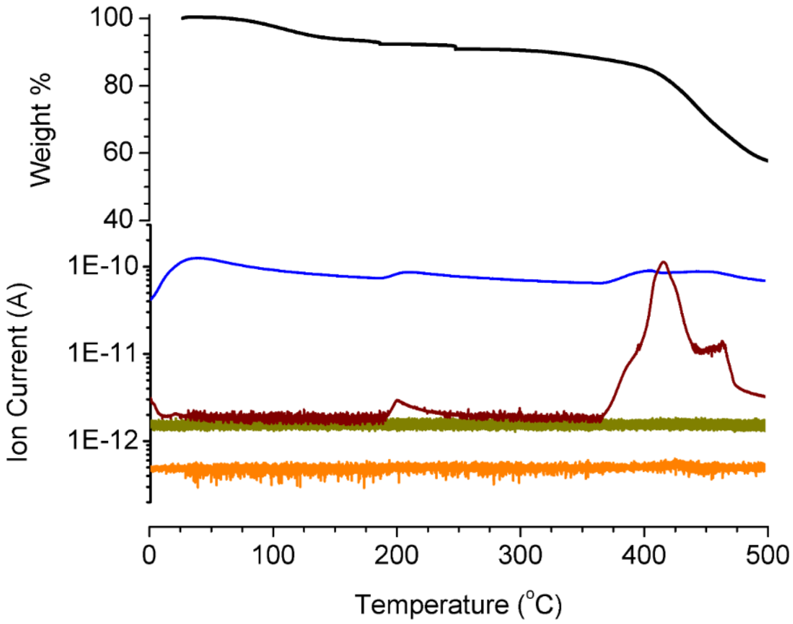

Figure S11. TGA curve(black) of the Fe/Zn MOF after thermal cycling where specific gases, H_2O (18amu blue), MeOH (31 amu gold), CO_2(44amu maroon), and DMF (73 amu orange), were monitored for the temperature of gas release from the sample.

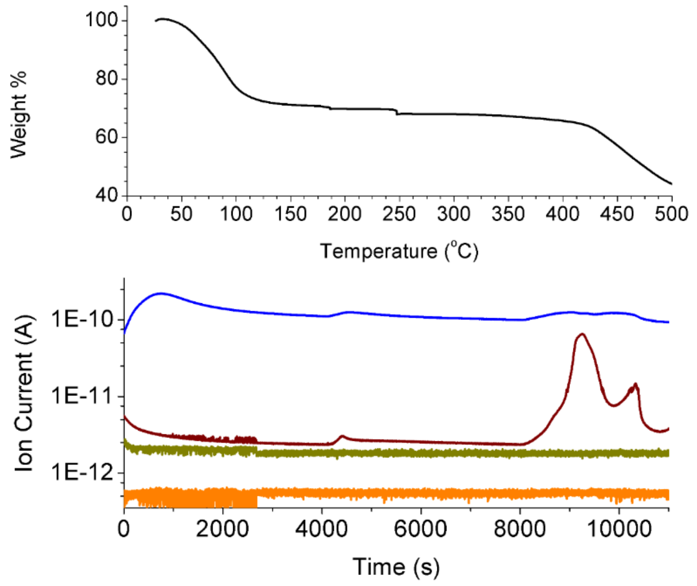

Figure S12. TGA curve(black) of the Fe/Mn MOF before thermal cycling where specific gases, H_2O (18amu blue), MeOH (31 amu gold), CO_2(44amu maroon), and DMF (73 amu orange), were monitored for the temperature of gas release from the sample.

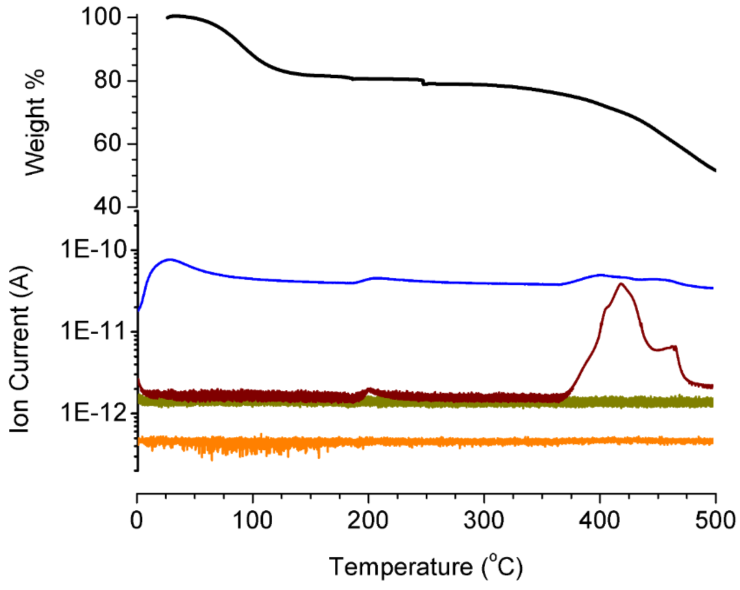

Figure S13. TGA curve(black) of the Fe/Mn MOF after thermal cycling where specific gases, H_2O (18amu blue), MeOH (31 amu gold), CO_2(44amu maroon), and DMF (73 amu orange), were monitored for the temperature of gas release from the sample.

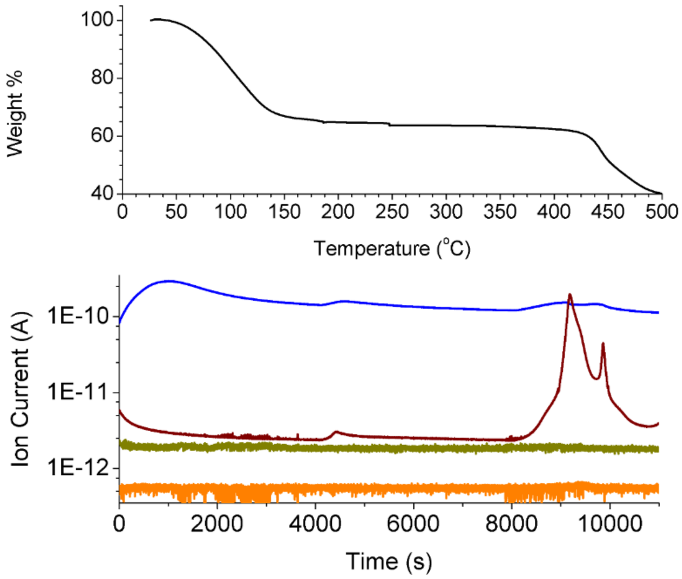

Figure S14. TGA curve(black) of the Fe/Co MOF before thermal cycling where specific gases, H_2O (18amu blue), MeOH (31 amu gold), CO_2(44amu maroon), and DMF (73 amu orange), were monitored for the temperature of gas release from the sample.

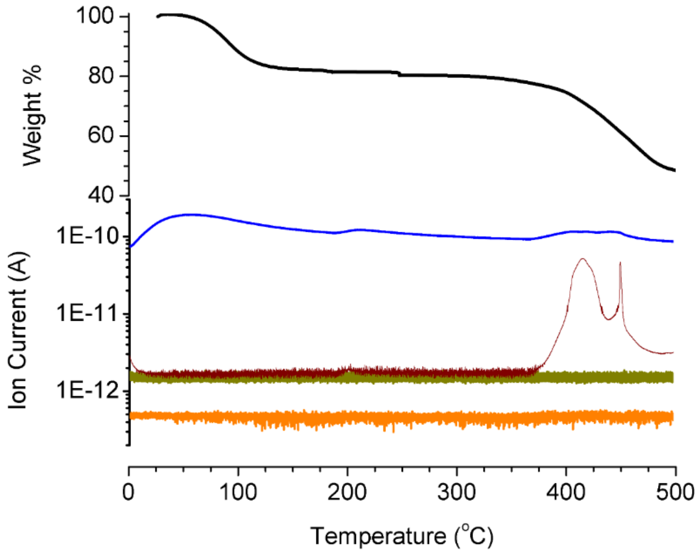

Figure S15. TGA curve(black) of the Fe/Co MOF after thermal cycling where specific gases, H_2O (18amu blue), MeOH (31 amu gold), CO_2(44amu maroon), and DMF (73 amu orange), were monitored for the temperature of gas release from the sample.

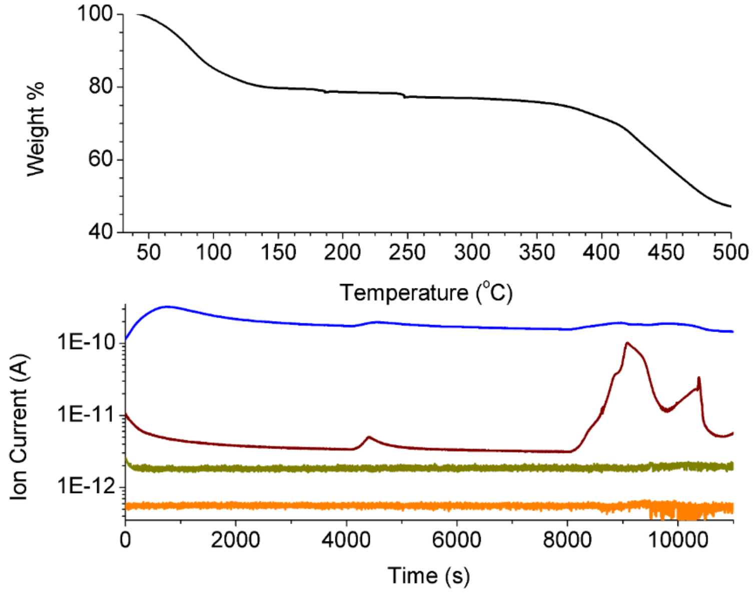

Figure S16. TGA curve(black) of the Fe_3 MOF before thermal cycling where specific gases, H_2O (18amu blue), MeOH (31 amu gold), CO_2(44amu maroon), and DMF (73 amu orange), were monitored for the temperature of gas release from the sample.

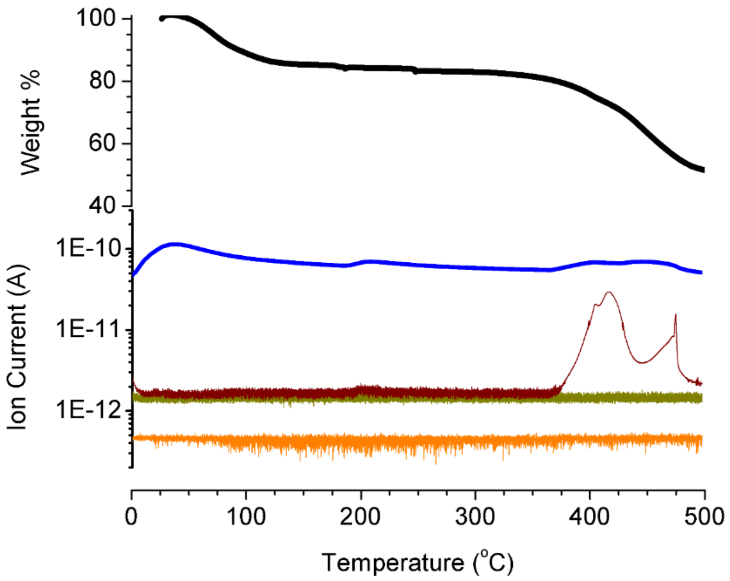

Figure S17. TGA curve(black) of the Fe_3 MOF after thermal cycling where specific gases, H_2O (18amu blue), MeOH (31 amu gold), CO_2(44amu maroon), and DMF (73 amu orange), were monitored for the temperature of gas release from the sample.

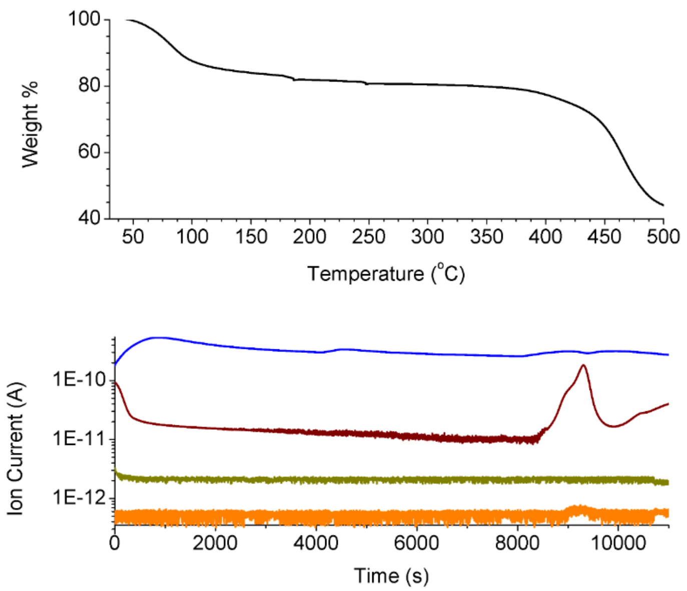

Figure S18. TGA curve(black) of the In_3 MOF before thermal cycling where specific gases, H_2O (18amu blue), MeOH (31 amu gold), CO_2(44amu maroon), and DMF (73 amu orange), were monitored for the temperature of gas release from the sample.

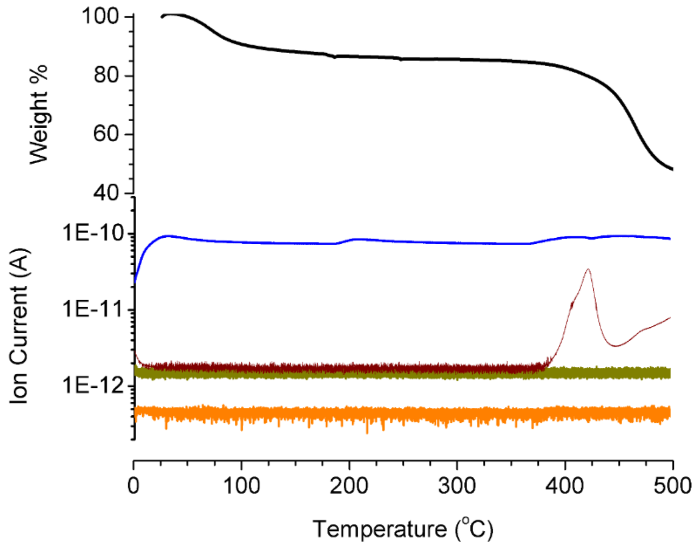

Figure S19. TGA curve(black) of the In_3 MOF after thermal cycling where specific gases, H_2O (18amu blue), MeOH (31 amu gold), CO_2(44amu maroon), and DMF (73 amu orange), were monitored for the temperature of gas release from the sample.

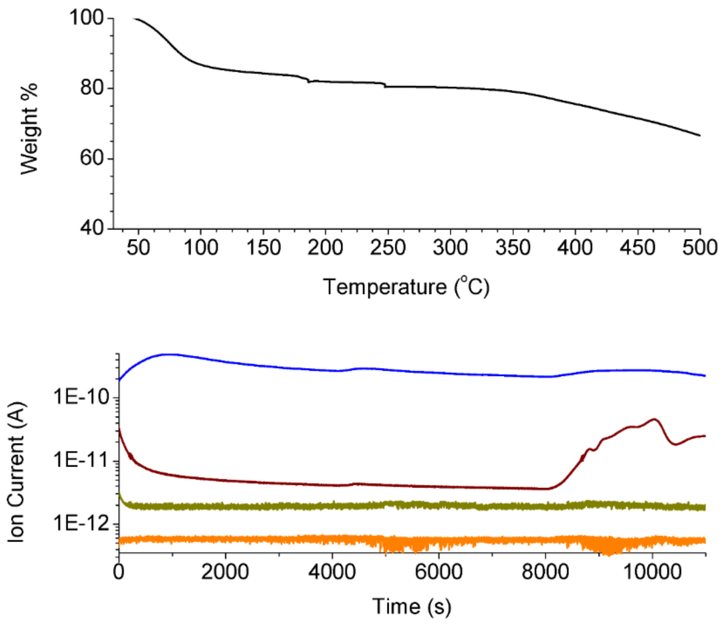

Figure S20. TGA curve(black) of the Sc_3 MOF before thermal cycling where specific gases, H_2O (18amu blue), MeOH (31 amu gold), CO_2(44amu maroon), and DMF (73 amu orange), were monitored for the temperature of gas release from the sample.

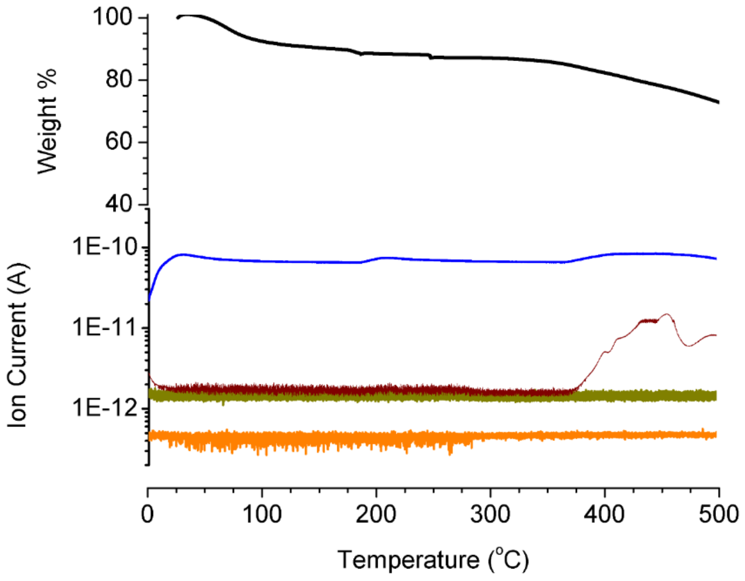

Figure S21. TGA curve(black) of the Sc_3 MOF after thermal cycling where specific gases, H_2O (18amu blue), MeOH (31 amu gold), CO_2(44amu maroon), and DMF (73 amu orange), were monitored for the temperature of gas release from the sample.

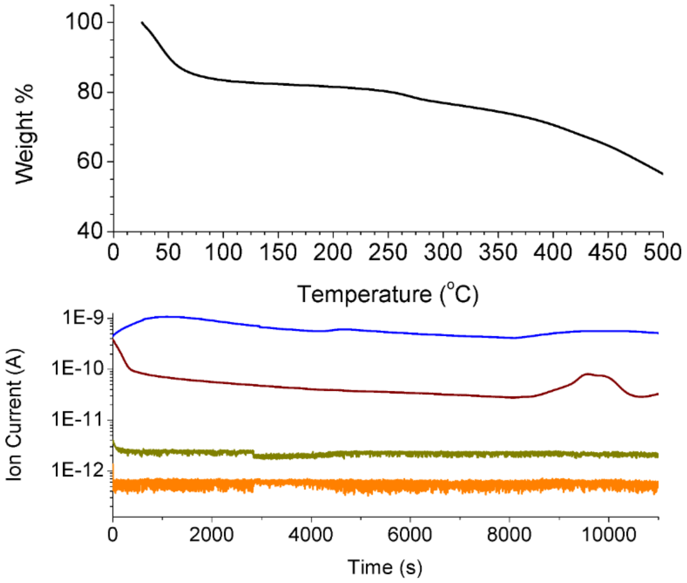

Figure S22. TGA curve(black) of the Al$_3$ MOF before thermal cycling where specific gases, H_2O (18amu blue), MeOH (31 amu gold), CO_2(44amu maroon), and DMF (73 amu orange), were monitored for the temperature of gas release from the sample.

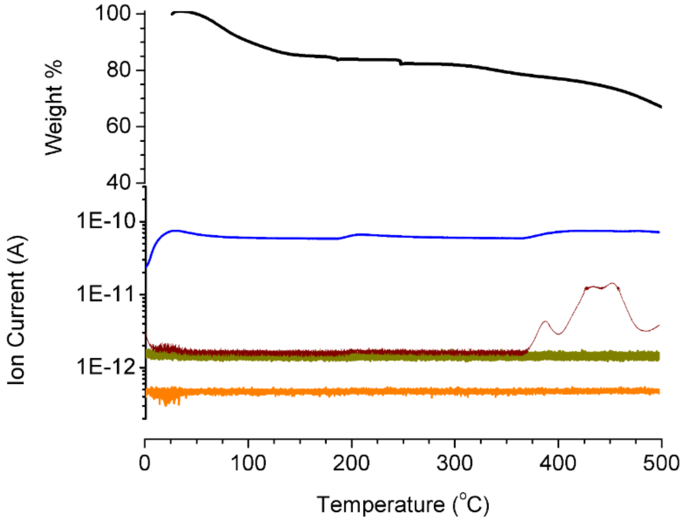

Figure S23. TGA curve(black) of the Al$_3$ MOF after thermal cycling where specific gases, H_2O (18amu blue), MeOH (31 amu gold), CO_2(44amu maroon), and DMF (73 amu orange), were monitored for the temperature of gas release from the sample.

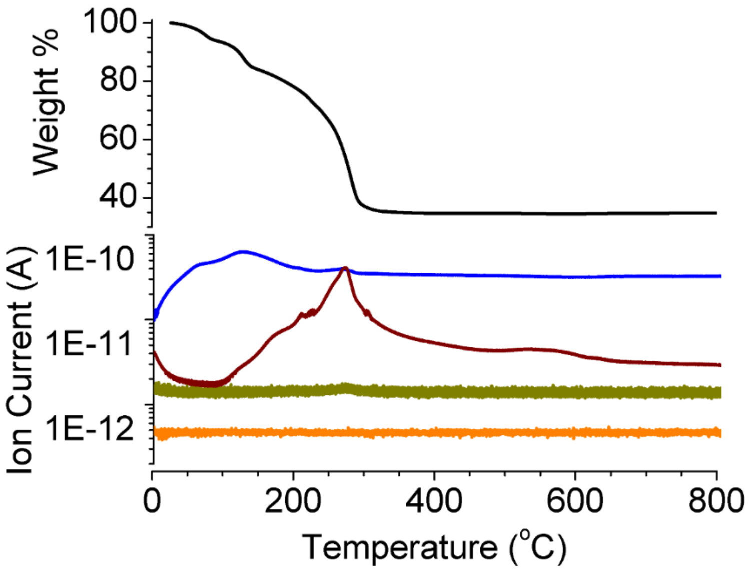

Figure S24. TGA curve (black) of the Fe/Zn preformed cluster where specific gases, H_2O (18amu blue), MeOH (31 amu gold), CO_2(44amu maroon), and DMF (73 amu orange), were monitored for the temperature of gas release from the sample.

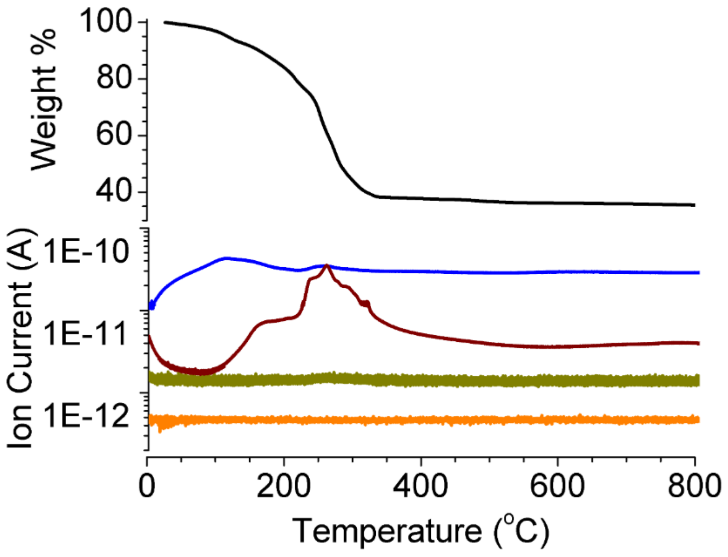

Figure S25. TGA curve(black) of the Fe_3 preformed cluster where specific gases, H_2O (18amu blue), MeOH (31 amu gold), CO_2(44amu maroon), and DMF (73 amu orange), were monitored for the temperature of gas release from the sample.

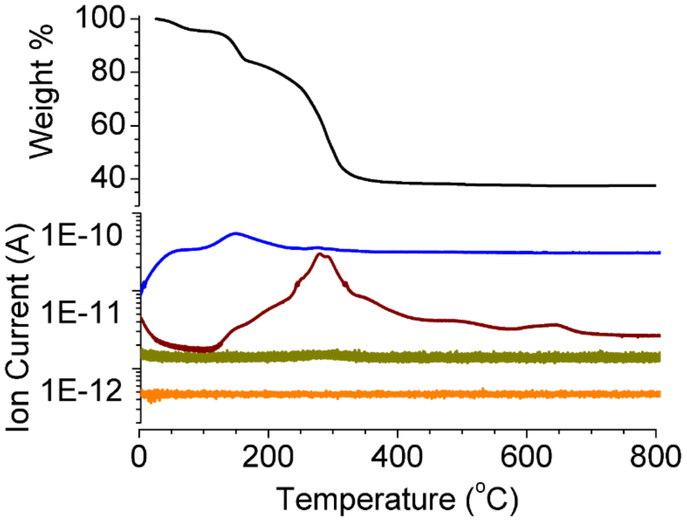

Figure S26. TGA curve(black) of the Fe/Ni preformed cluster where specific gases, H_2O (18amu blue), MeOH (31 amu gold), CO_2(44amu maroon), and DMF (73 amu orange), were monitored for the temperature of gas release from the sample.

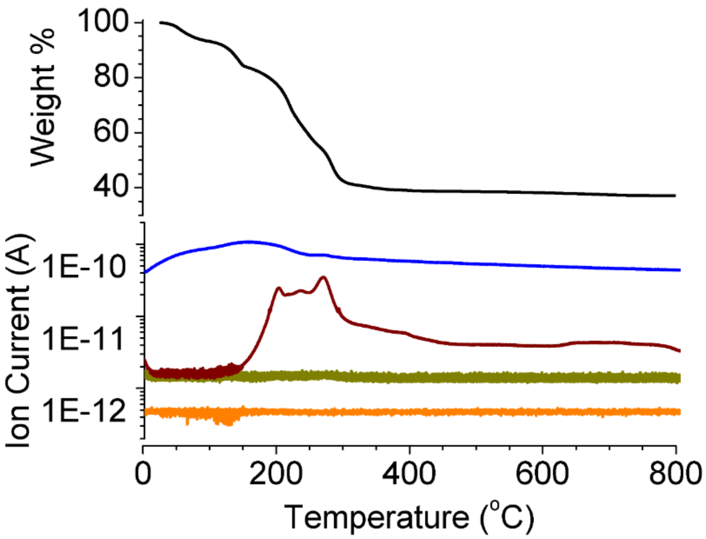

Figure S27. TGA curve(black) of the Fe/Mn preformed cluster where specific gases, H_2O (18amu blue), MeOH (31 amu gold), CO_2(44amu maroon), and DMF (73 amu orange), were monitored for the temperature of gas release from the sample.

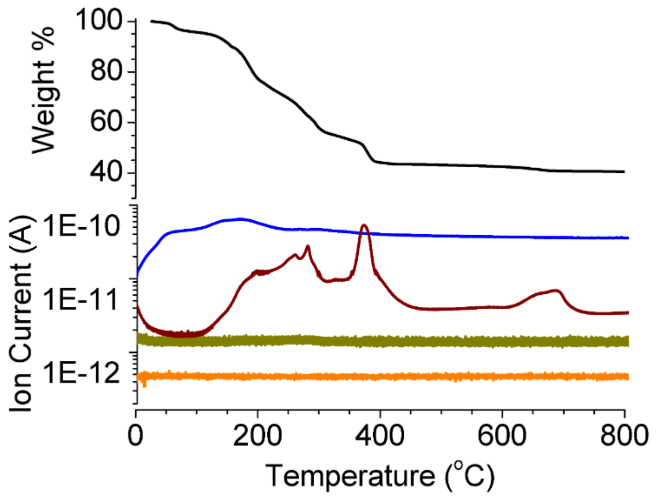

Figure S28. TGA curve(black) of the Fe/Co preformed cluster where specific gases, H_2O (18amu blue), MeOH (31 amu gold), CO_2(44amu maroon), and DMF (73 amu orange), were monitored for the temperature of gas release from the sample.

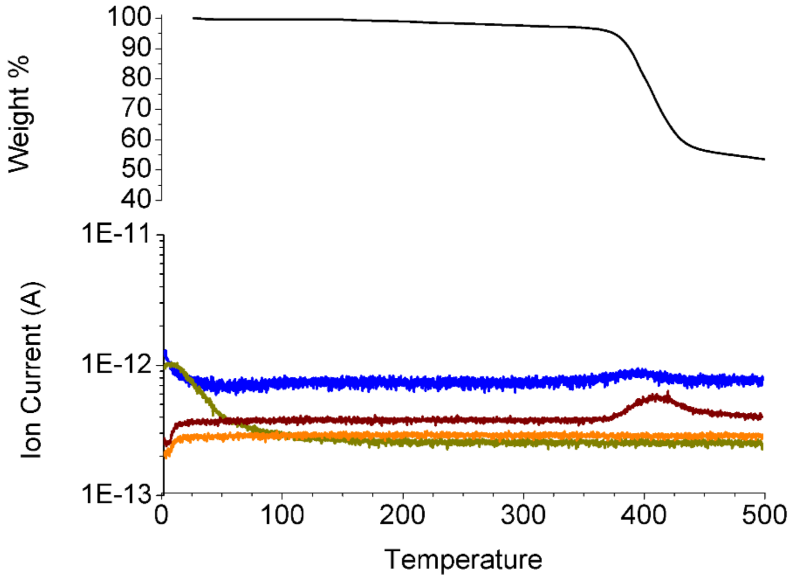

Figure S29. TGA curve(black) of the ABTC linker where specific gases, H_2O (18amu blue), MeOH (31 amu gold), CO_2(44amu maroon), and DMF (73 amu orange), were monitored for the temperature of gas release from the sample.

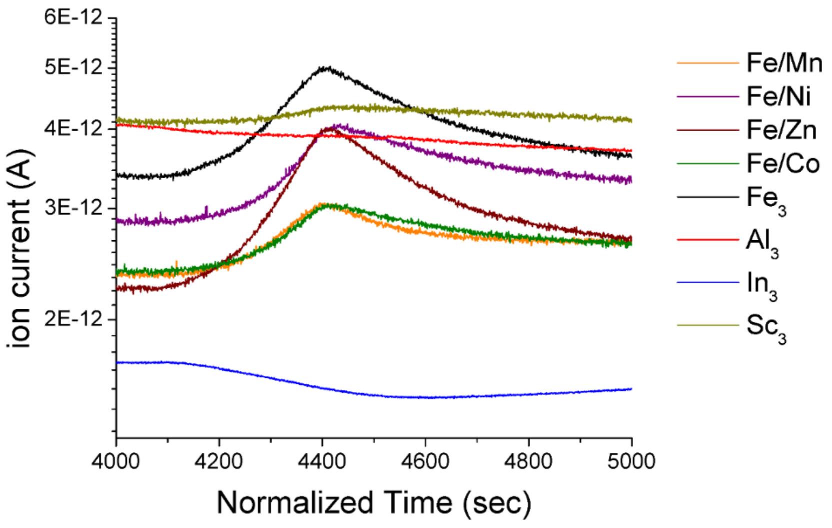

Figure S30. CO_2 evolution during TGA-MS studies for all samples. The MS data has been normalized to time as decarboxylation occur at slightly different temperatures for the redox active cluster containing MOFs. CO_2 evolution at decarboxylation is only observable for the redox active cluster containing MOFs (Fe/Co, Fe/Mn, Fe/Ni, Fe/Zn, and Fe_3).

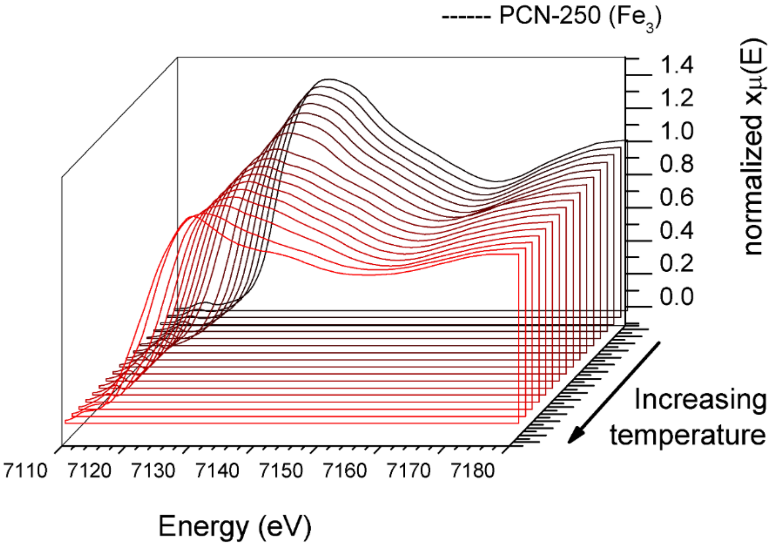

Figure S31. XAS of PCN-250 (Fe_3) during heating study from 23 °C to 450 °C in the range of 7110 eV to 7180 eV.

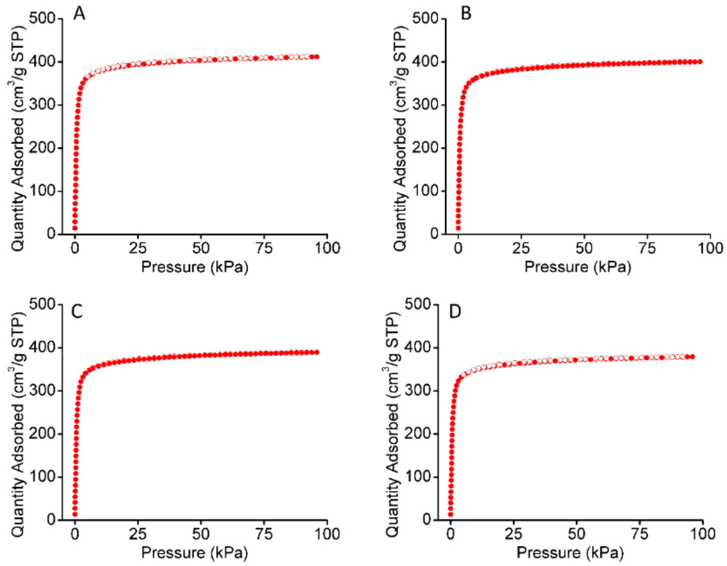

Figure S32. N_2 Isotherms taken at 77K before samples underwent 100 hours of 185 °C vacuum activation. A) PCN250 (Fe/Zn), B) PCN-250 (Fe/Mn), C) PCN-250 (Fe/Co), D) PCN-250 (Fe/Ni). All of the samples show a characteristic type 1 isotherm.[6]

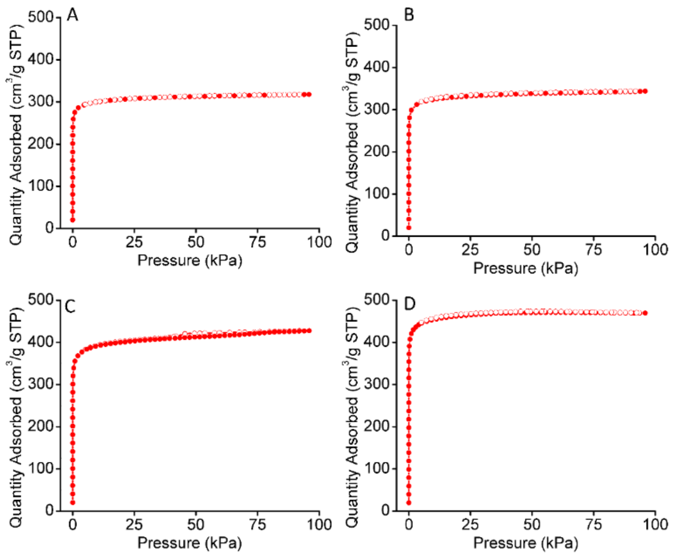

Figure S33. N_2 Isotherms taken at 77K before samples underwent 100 hours of 185 °C vacuum activation. A) PCN250 (Sc_3), B) PCN-250 (In_3), C) PCN-250 (Fe_3), D) PCN-250 (Al_3). All of the samples show a characteristic type 1 isotherm.[6]

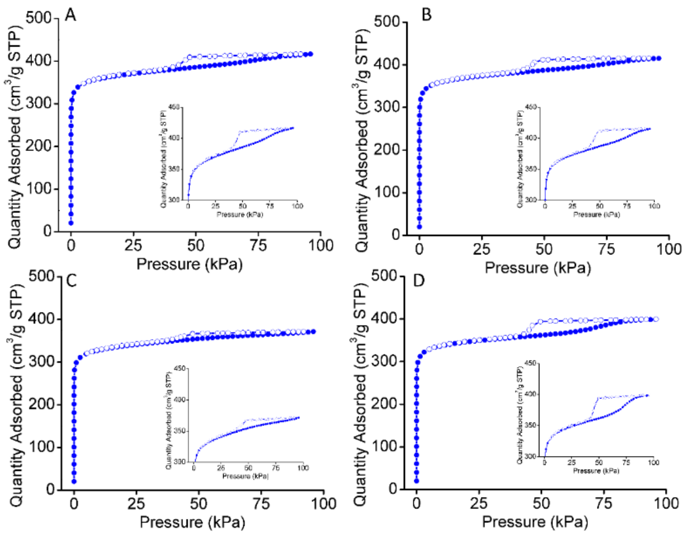

Figure S34. N_2 Isotherms taken at 77K after samples underwent 100 hours of 185 °C vacuum activation. A) PCN250 (Fe/Co), B) PCN-250 (Fe/Mn), C) PCN-250 (Fe/Ni), D) PCN-250 (Fe/Zn). All of the samples of the mixed metal cluster containing MOFs have generated mesopores as characterized by hysteresis loop formation.[6]

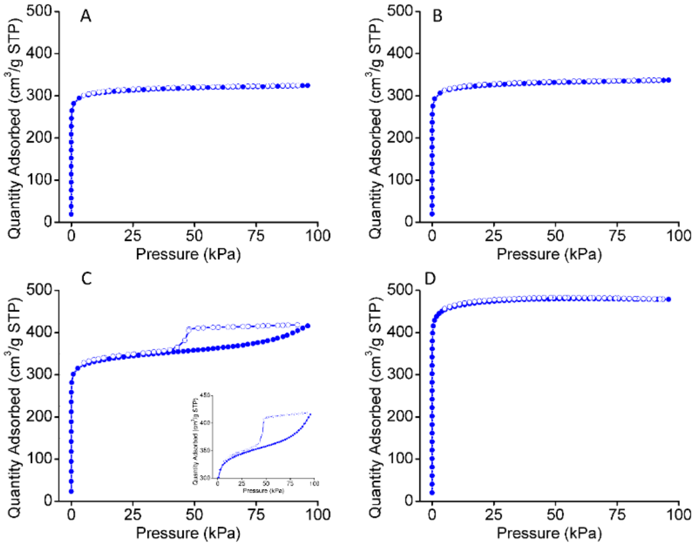

Figure S35. N_2 Isotherms taken at 77K after samples underwent 100 hours of 185 °C vacuum activation. A) PCN-250 (In_3), B) PCN-250 (Sc_3), C) PCN-250 (Fe_3), D) PCN-250 (Al_3). Only The PCN-250 (Fe_3) sample shows mesopore generation as characterized by hysteresis loop formation.[6]

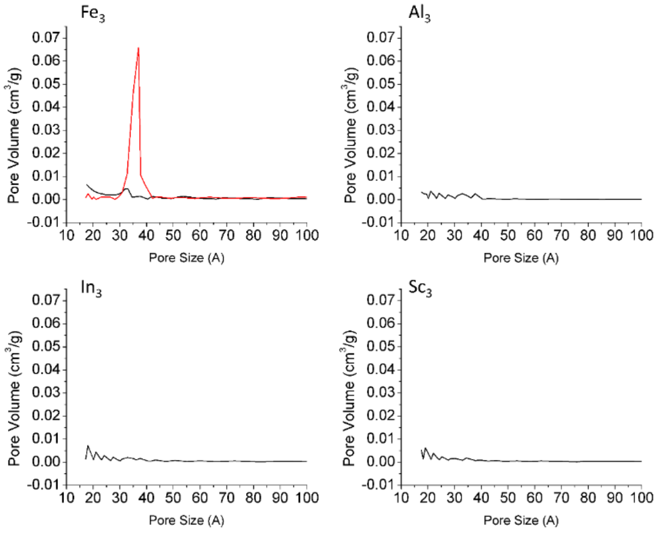

Figure S36. Pore Size distribution as compared to Pore Volume (cm^3/g) in the PCN250 Isomorphs. The microporous samples(black) show no evidence of the 37Å mesopore that is formed during decarboxylation. The mesoporous samples (red) show the presence of the mesopore at 37Å. This pore is the result of cluster defect losses as the result of thermal decarboxylation.

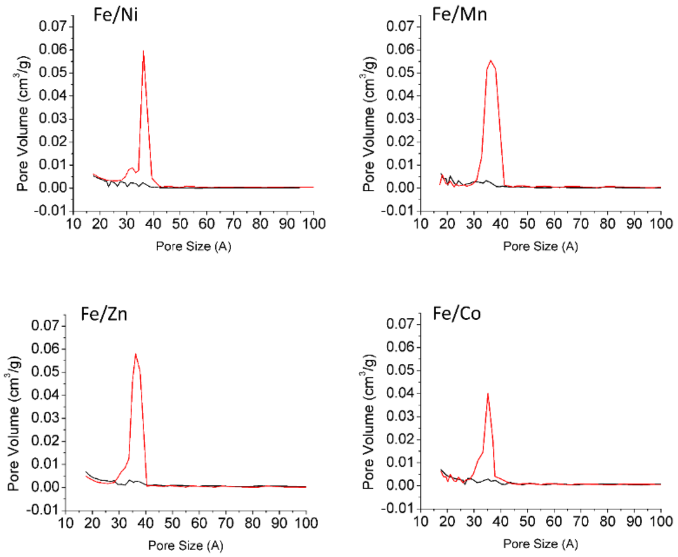

Figure S37. Pore Size distribution as compared to Pore Volume (cm^3/g) in the PCN250 Isomorphs. The microporous samples(black) show no evidence of the 37Å mesopore that is formed during decarboxylation. The mesoporous samples (red) show the presence of the mesopore at 37Å. This pore is the result of cluster defect losses as the result of thermal decarboxylation.

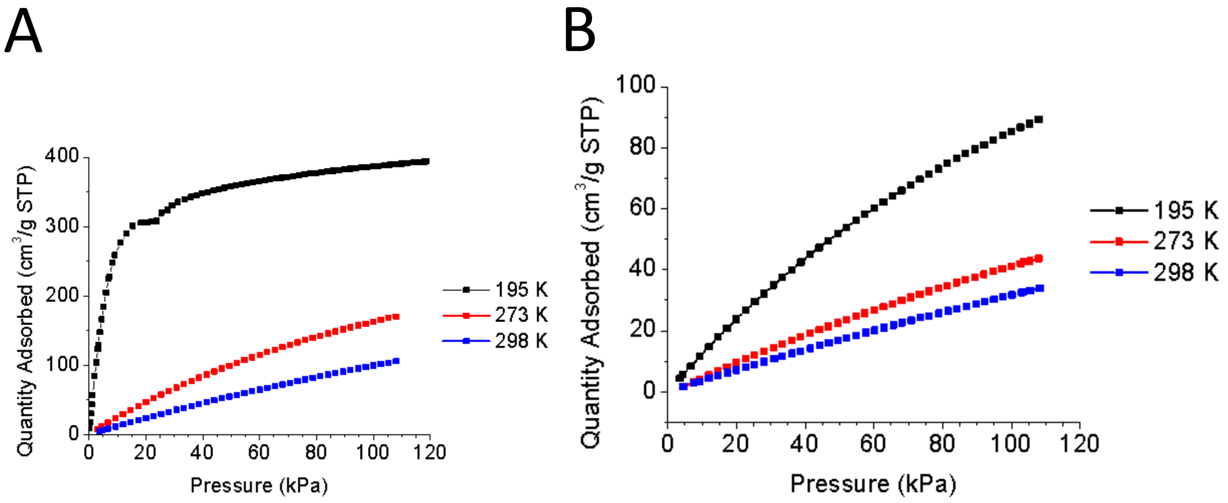

Figure S38. Adsorption Isotherms of PCN-250 (Al_3) for A) CO_2 and B) CH_4.

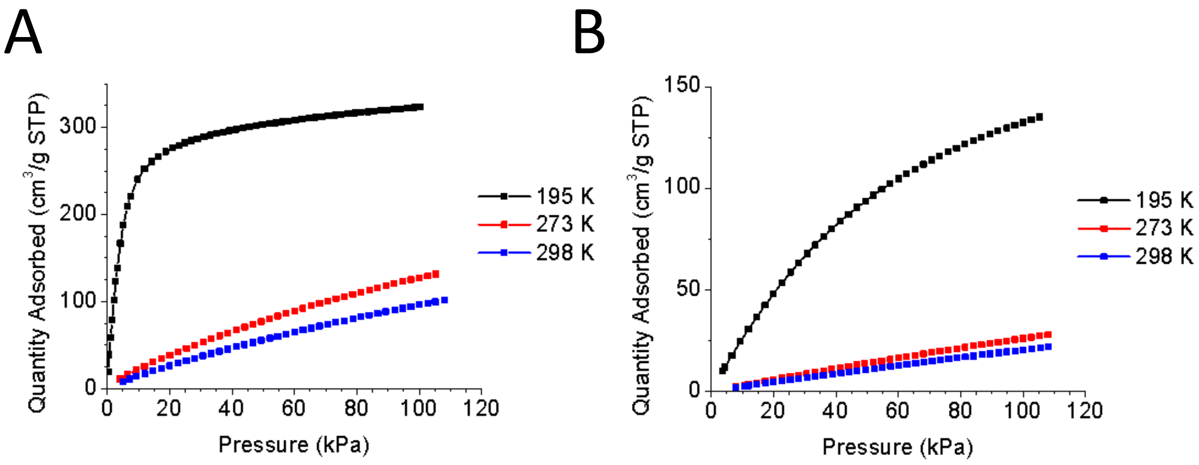

Figure S39. Adsorption Isotherms of PCN-250 (Fe_3) meso for A) CO_2 and B) CH_4.

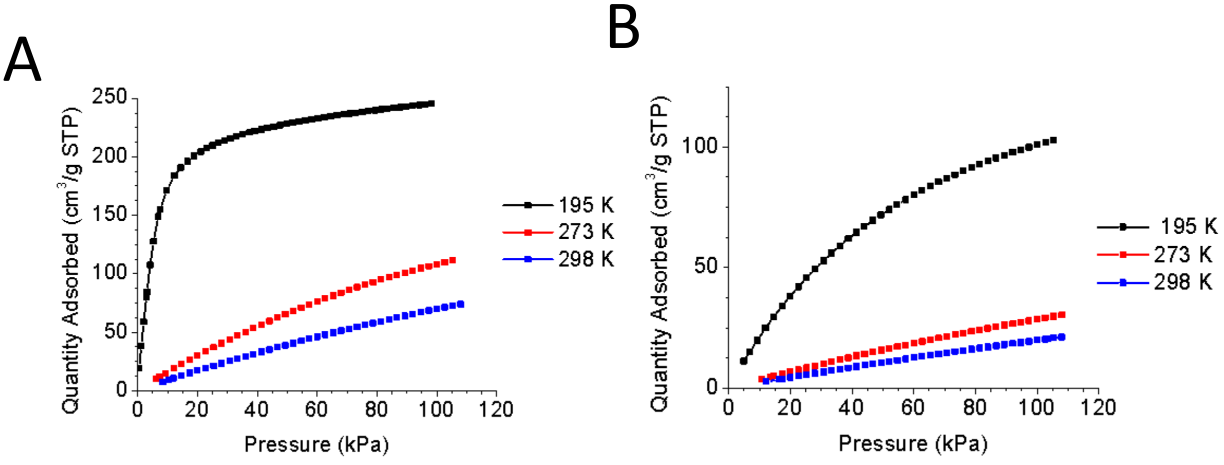

Figure S40. Adsorption Isotherms of PCN-250 (Fe_3) micro for A) CO_2 and B) CH_4.

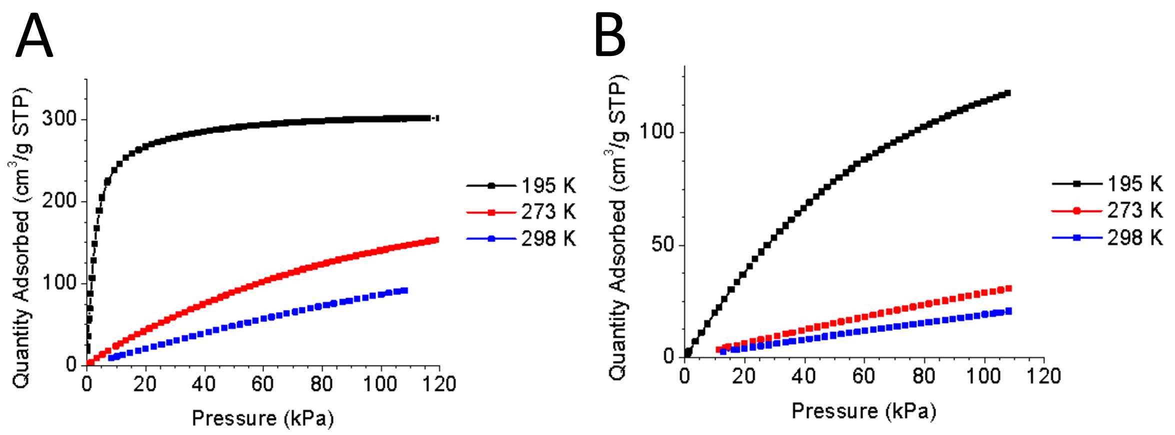

Figure S41. Adsorption Isotherms of PCN-250 (Fe/Zn) micro for A) CO_2 and B) CH_4.

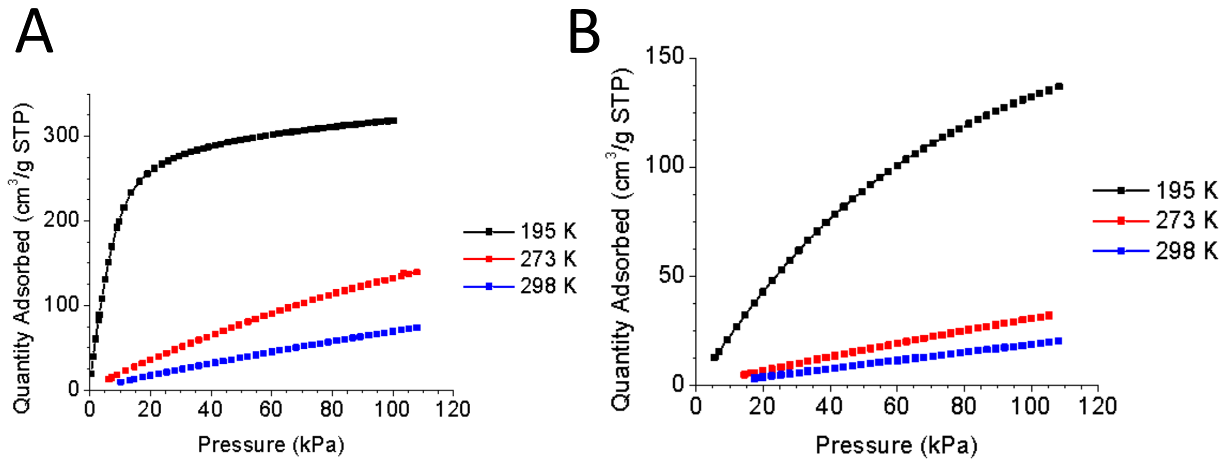

Figure S42. Adsorption Isotherms of PCN-250 (Fe/Ni) micro for A) CO_2 and B) CH_4.

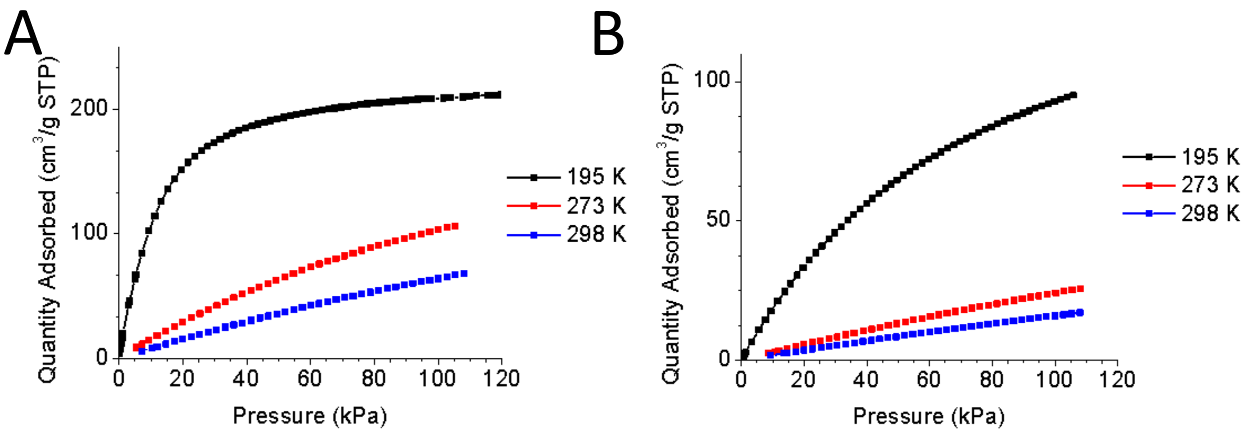

Figure S43. Adsorption Isotherms of PCN-250 (In) micro for A) CO_2 and B) CH_4.

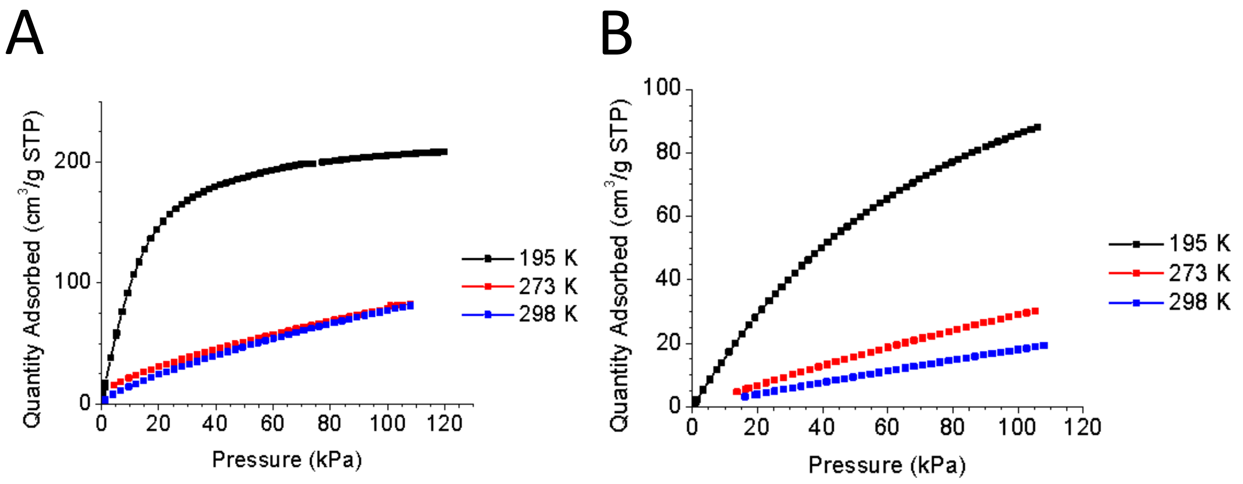

Figure S44. Adsorption Isotherms of PCN-250 (Sc) micro for A) CO_2 and B) CH_4.

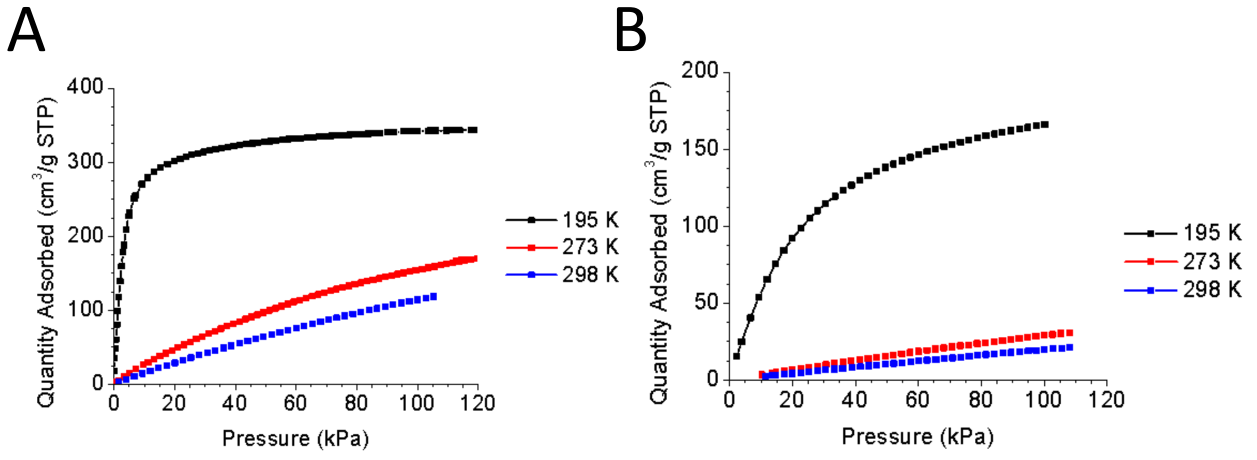

Figure S45. Adsorption Isotherms of PCN-250 (Fe/Co) meso for A) CO_2 and B) CH_4.

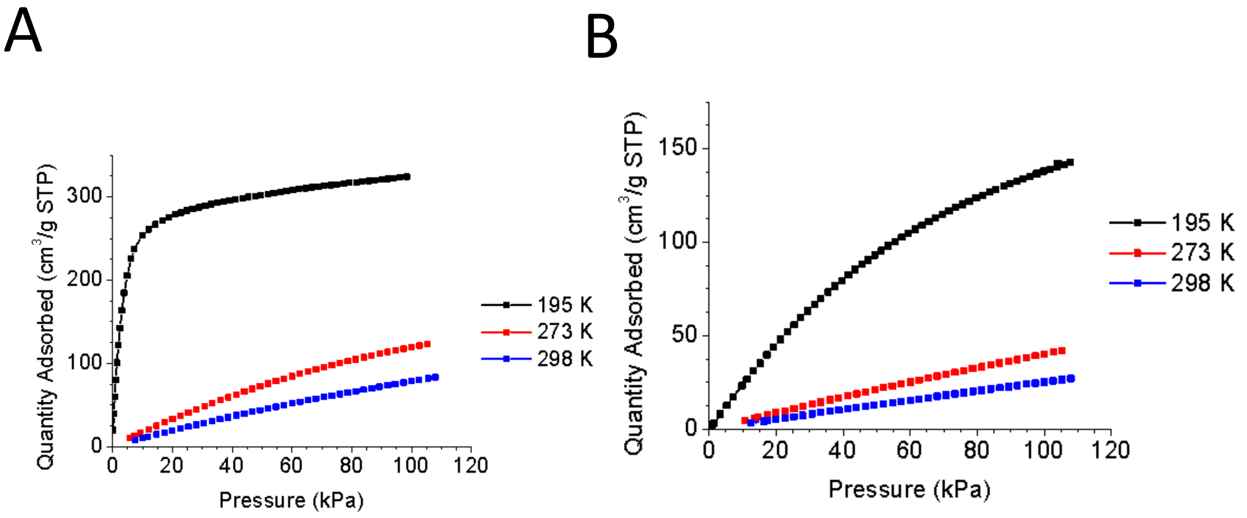

Figure S46. Adsorption Isotherms of PCN-250 (Fe/Co) micro for A) CO_2 and B) CH_4.

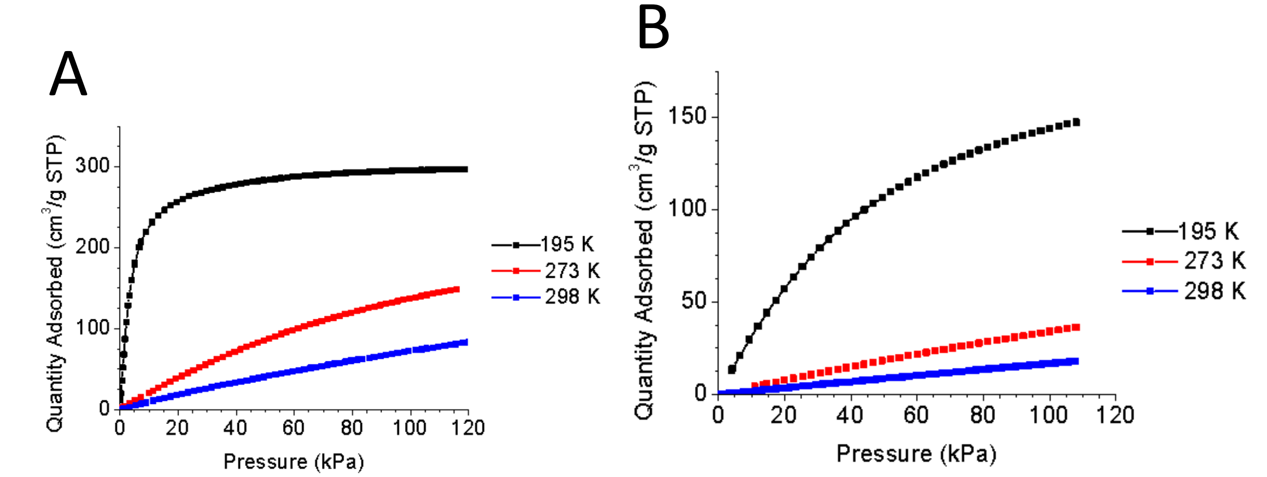

Figure S47. Adsorption Isotherms of PCN-250 (Fe/Mn) meso for A) CO_2 and B) CH_4.

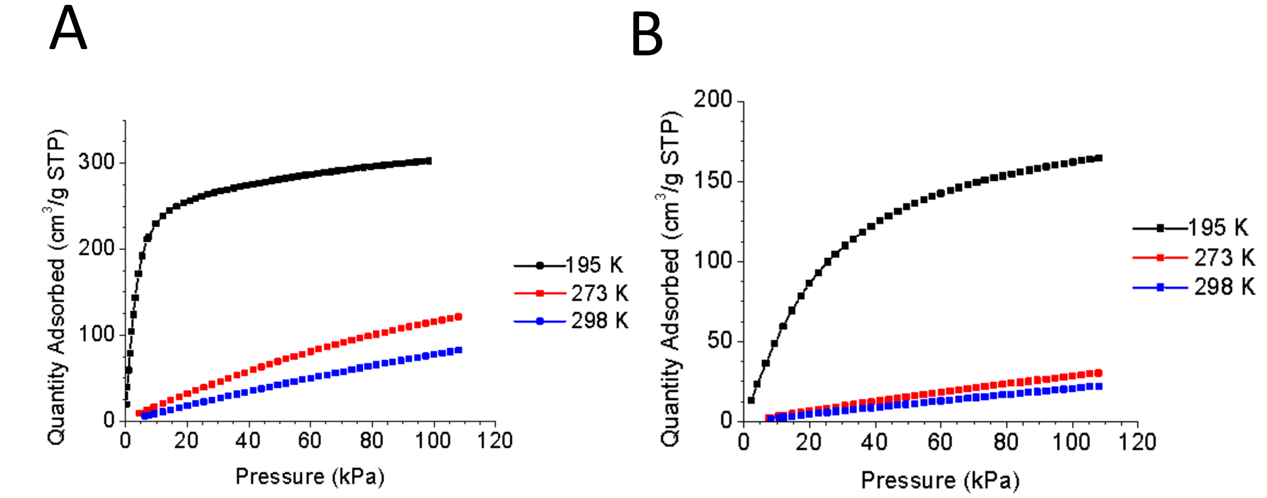

Figure S48. Adsorption Isotherms of PCN-250 (Fe/Mn) micro for A) CO_2 and B) CH_4.

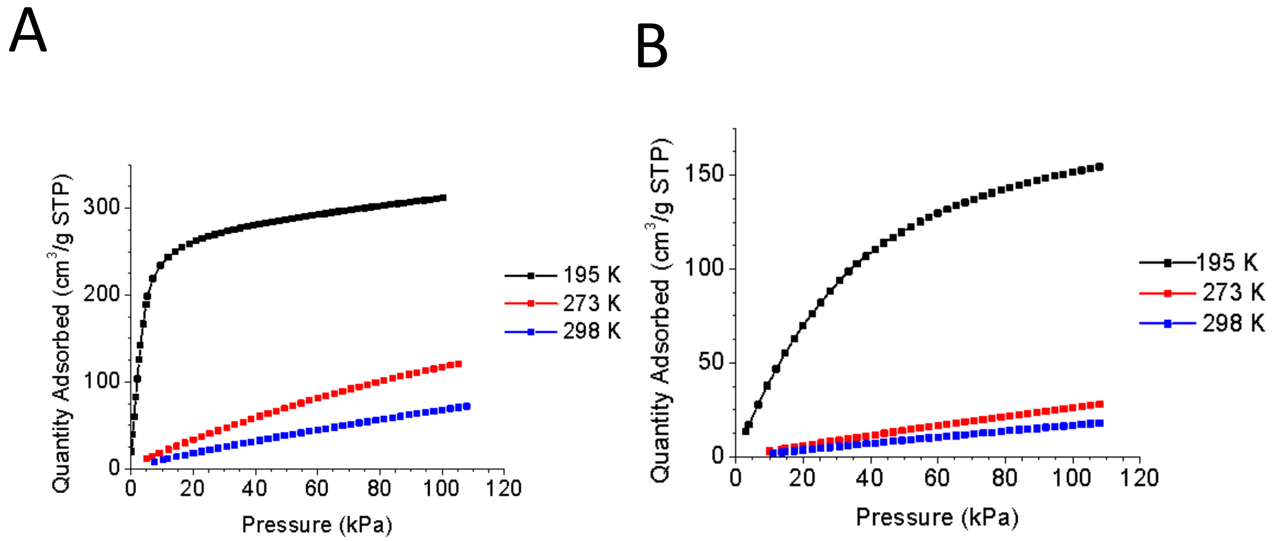

Figure S49. Adsorption Isotherms of PCN-250 (Fe/Ni) meso for A) CO_2 and B) CH_4.

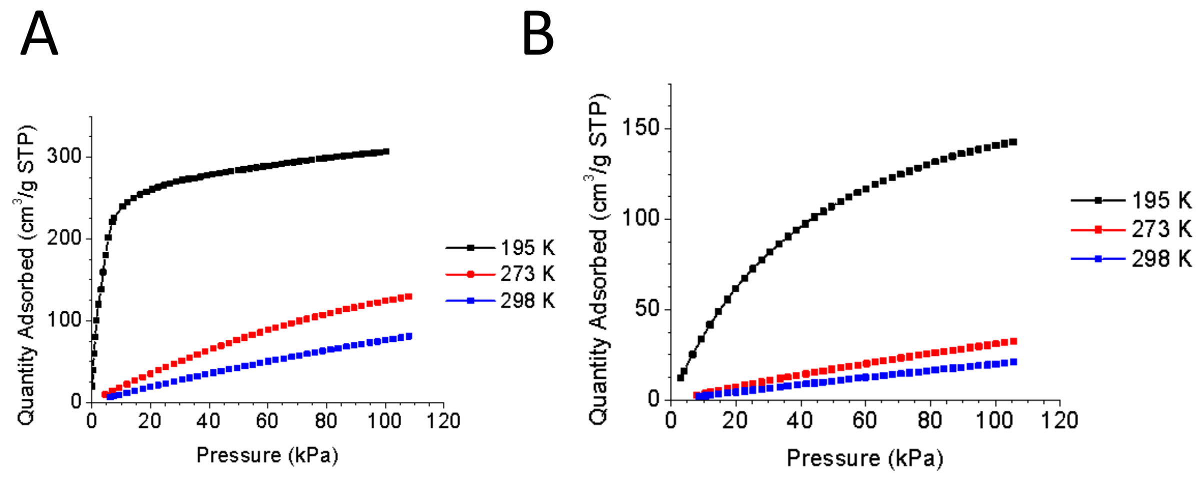

Figure S50. Adsorption Isotherms of PCN-250 (Fe/Zn) meso for A) CO_2 and B) CH_4.

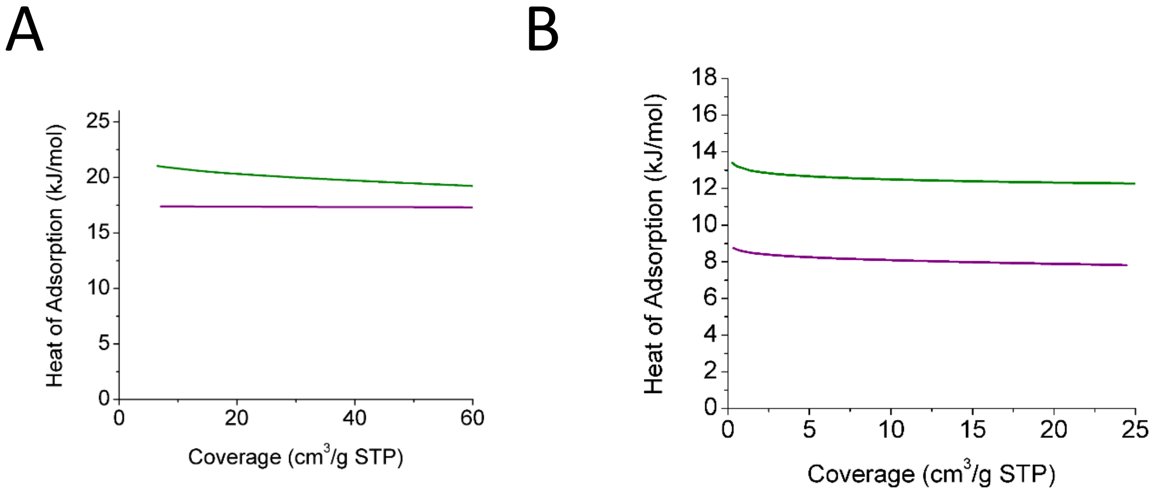

Figure S51. Heat of Adsorptions calculated for PCN-250 (Fe/Ni) A) CO_2 and B) CH_4. Microporous sample (purple), Mesoporous sample (green).

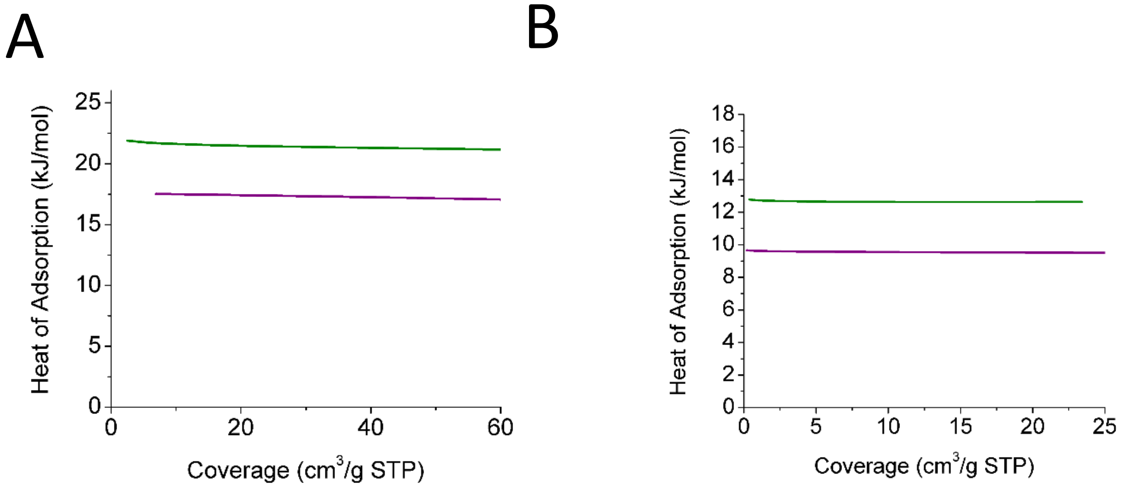

Figure S52. Heat of Adsorptions calculated for PCN-250 (Fe/Zn) A) CO_2 and B) CH_4. Microporous sample (purple), Mesoporous sample (green).

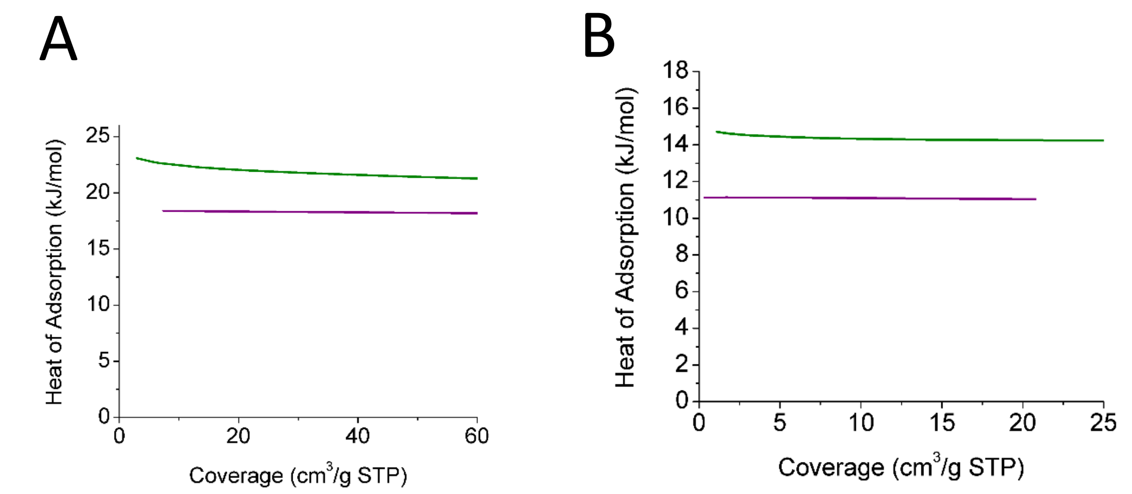

Figure S53. Heat of Adsorptions calculated for PCN-250 (Fe/Mn) A) CO_2 and B) CH_4. Microporous sample (purple), Mesoporous sample (green).

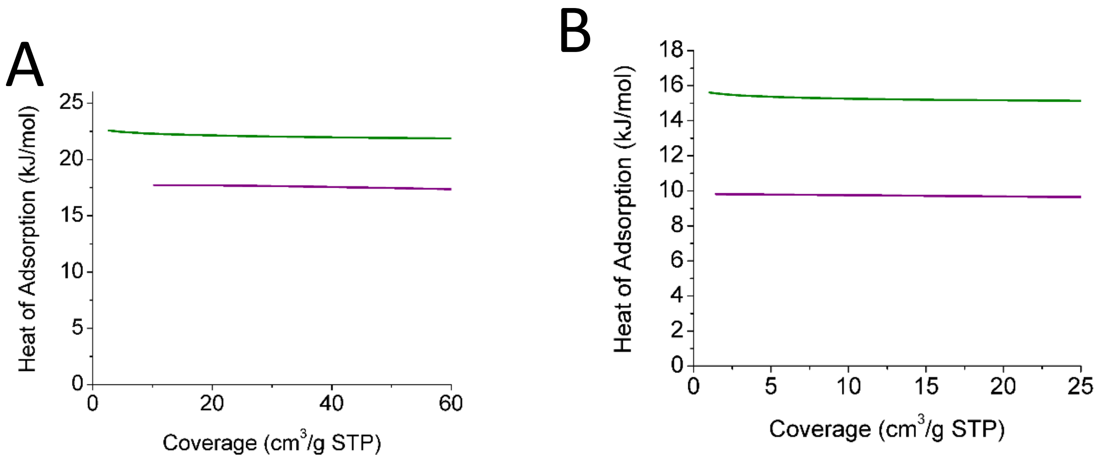

Figure S54. Heat of Adsorptions calculated for PCN-250 (Fe/Co) A) CO_2 and B) CH_4. Microporous sample (purple), Mesoporous sample (green).

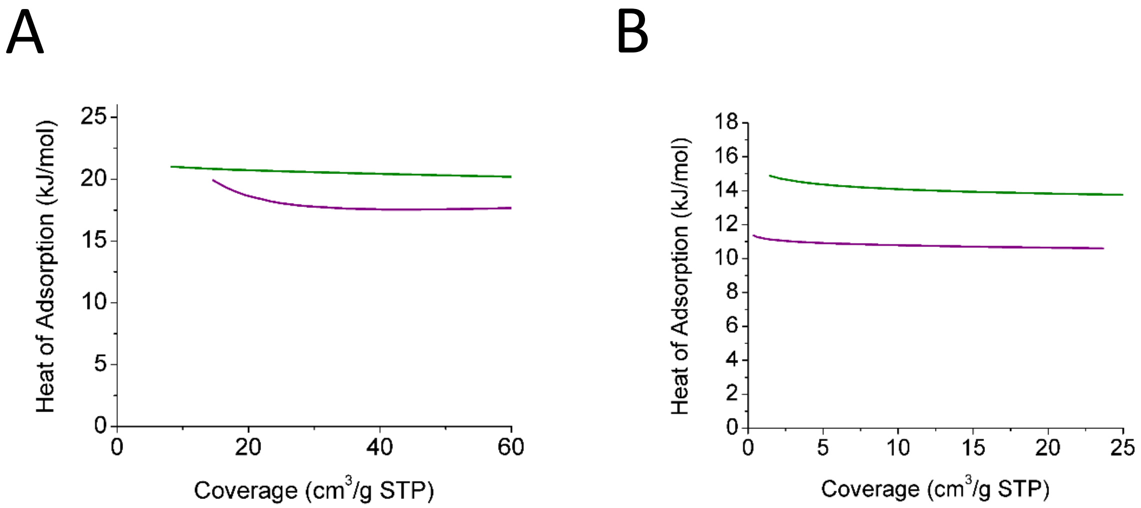

Figure S55. Heat of Adsorptions calculated for PCN-250 (Fe_3) A) CO_2 and B) CH_4. Microporous sample (purple), Mesoporous sample (green).

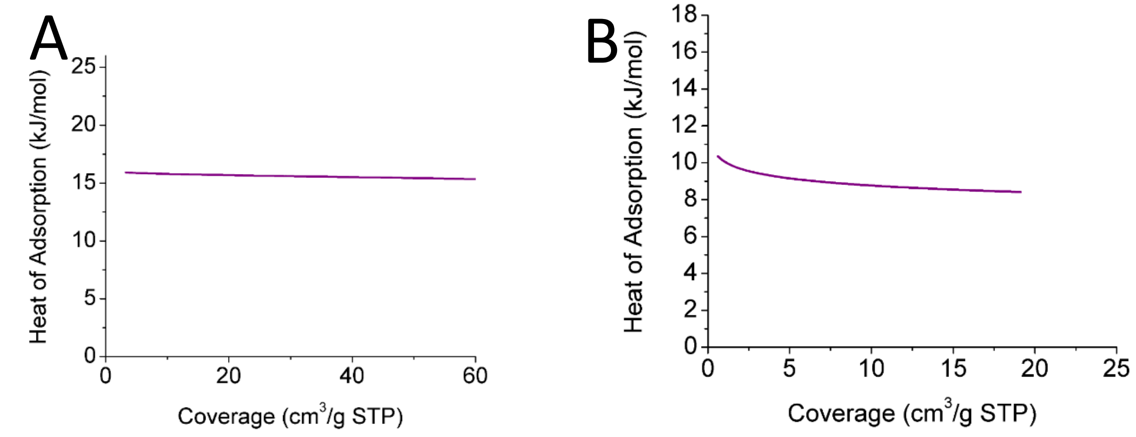

Figure S56. Heat of Adsorptions calculated for PCN-250 (In_3) A) CO_2 and B) CH_4. Microporous sample (purple), No Mesoporous samples of this compound were able to be generated using the thermal decarboxylation method.

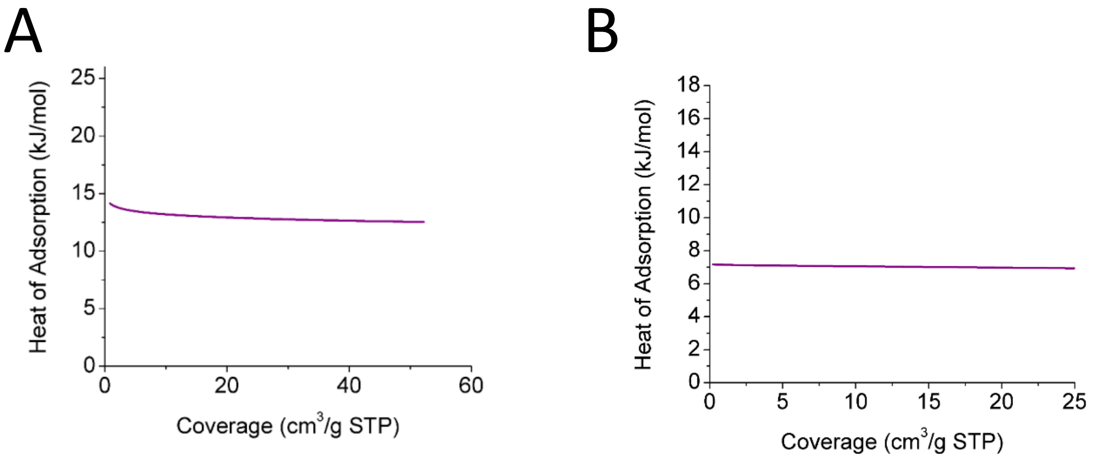

Figure S57. Heat of Adsorptions calculated for PCN-250 (Sc_3) A) CO_2 and B) CH_4. Microporous sample (purple), No Mesoporous samples of this compound were able to be generated using the thermal decarboxylation method.

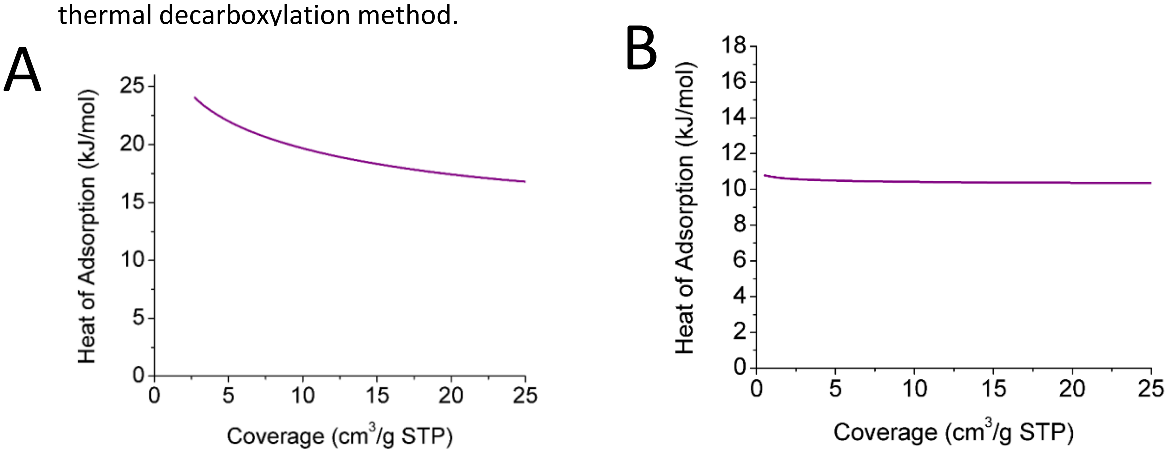

Figure S58. Heat of Adsorptions calculated for PCN-250 (Al_3) A) CO_2 and B) CH_4. Microporous sample (purple), No Mesoporous samples of this compound were able to be generated using the thermal decarboxylation method.

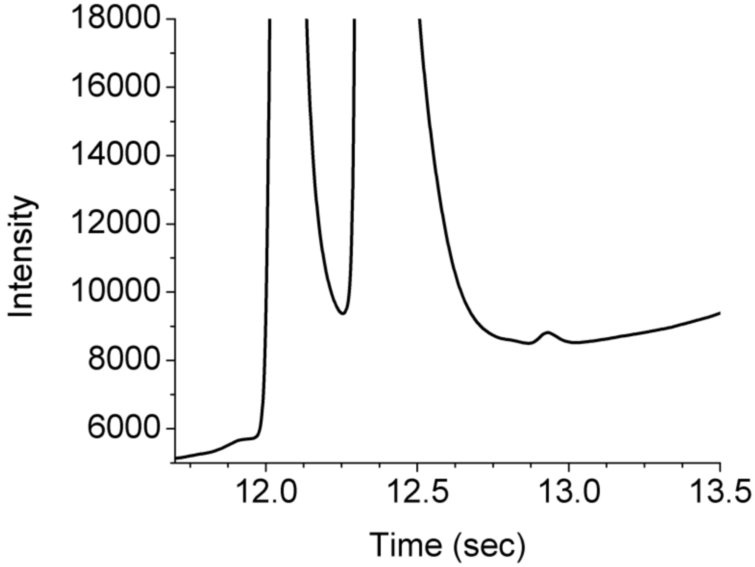

Figure S59. UV-GC Chromatogram of the decomposed sample of PCN-250 (Fe_3). The MOF was decomposed in pH=12 to yield the free H_4ABTC ligands in solution. The UV-GC Chromatagram was collected, yielding three peaks with each peak being sampled for the component MS spectrum. The two peaks at 12.16 sec and 12.50 sec match the mass of the H_4ABTC ligand. These two peaks correspond to the cis and trans form of H_4ABTC, as azobenzenes are known to undergo isomerization in the presence of UV light, with this isomerization changing the properties of the molecule.[7] The third peak at 13.06 sec matches the thermally decarboxylated ligand.

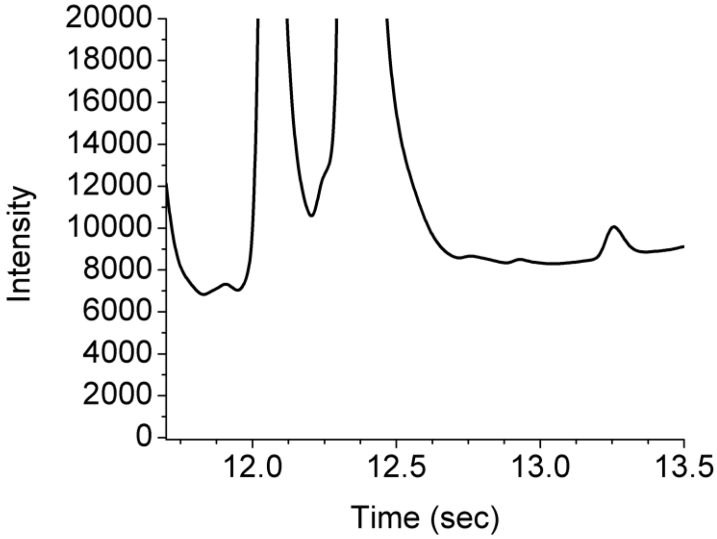

Figure S60. UV-GC Chromatogram of the decomposed sample, PCN-250 (Fe/Co). The MOF was decomposed in pH=12 to yield the free H_4ABTC ligands in solution. The UV-GC Chromatagram was collected yielding three peaks and each peak was sampled for its component MS spectrum. The two peaks at 12.17 sec and 12.48 sec match the mass of the H_4ABTC ligand. These two peaks correspond to the cis and trans form of H_4ABTC, as azobenzenes are known to undergo isomerization in the presence of UV light with this isomerization changing the properties of the molecule.[7] The third peak at 13.28 sec matches the thermally decarboxylated ligand.

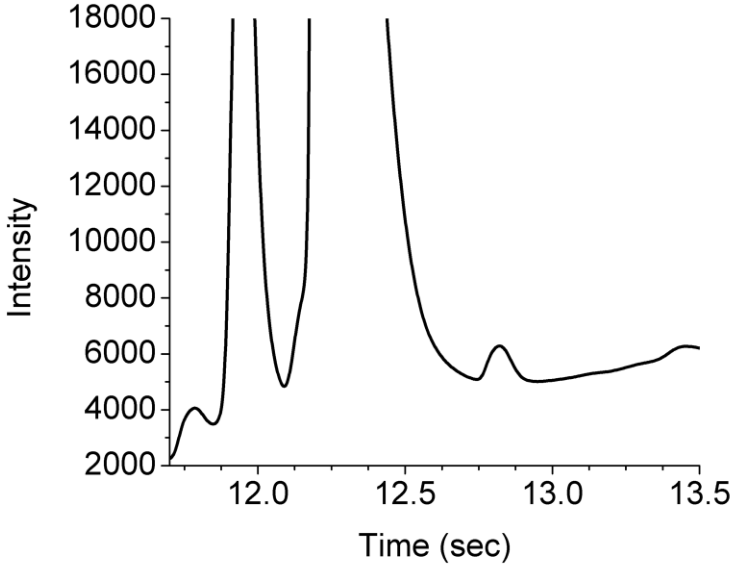

Figure S61. UV-GC Chromatogram of the decomposed sample of PCN-250 (Fe/Ni). The MOF was decomposed in pH=12 to yield the free H_4ABTC ligands in solution. The UV-GC Chromatagram was collected, yielding three peaks with each peak being sampled for their component MS spectrum. The two peaks at 11.92 sec and 12.39 sec match the mass of the H_4ABTC ligand. These two peaks correspond to the cis and trans form of H_4ABTC, as azobenzenes are known to undergo isomerization in the presence of UV light, with this isomerization changing the properties of the molecule.[7] The third peak at 12.87 sec matches the thermally decarboxylated ligand.

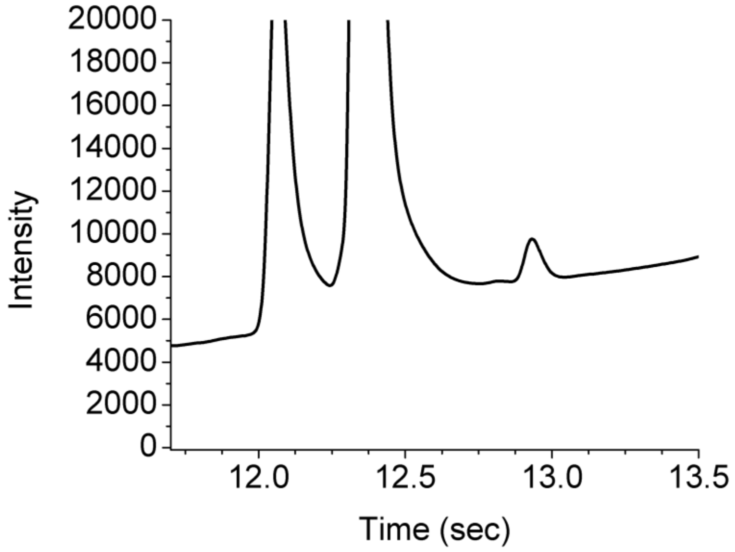

Figure S62. UV-GC Chromatogram of the decomposed sample of PCN-250 (Fe/Mn). The MOF was decomposed in pH=12 to yield the free H_4ABTC ligands in solution. The UV-GC Chromatagram was collected yielding three peaks, with each peak being sampled for their component MS spectrum. The two peaks at 12.13 sec and 12.46 sec match the mass of the H_4ABTC ligand. The two peaks present here correspond to the cis and trans form of H_4ABTC as azobenzenes are known to undergo isomerization in the presence of UV light, with this isomerization changing the properties of the molecule.[7] The third peak at 13.02 sec matches the thermally decarboxylated ligand.

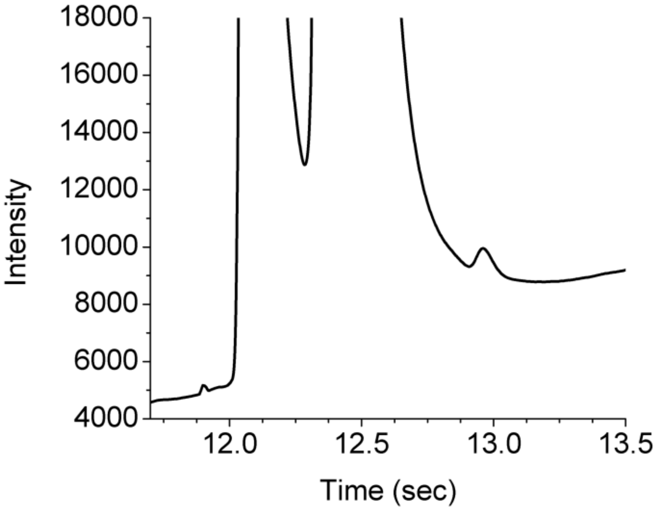

Figure S63. UV-GC Chromatogram of the decomposed sample of PCN-250 (Fe/Zn). The MOF was decomposed in pH=12 to yield the free H_4ABTC ligands in solution. The UV-GC Chromatagram was collected yielding three peaks, with each peak being sampled for their component MS spectrum. The two peaks at 12.12 sec and 12.53 sec match the mass of the H_4ABTC ligand. The two peaks present here correspond to the cis and trans form of H_4ABTC, as azobenzenes are known to undergo isomerization in the presence of UV light, with this isomerization changing the properties of the molecule.[7] The third peak at 13.01 sec matches the thermally decarboxylated ligand.

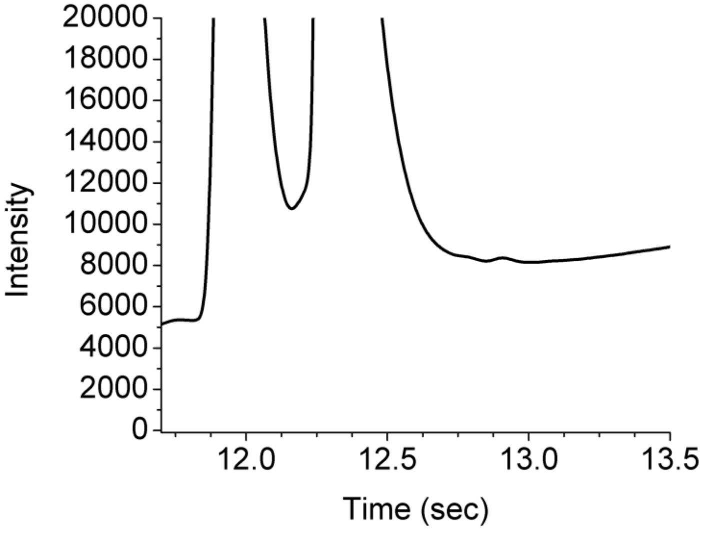

Figure S64. UV-GC Chromatogram of decomposed sample of PCN-250 (Sc_3). The MOF was decomposed in pH=12 to yield the free H_4ABTC ligands in solution. The UV-GC Chromatagram was collected yielding three peaks, with each peak being sampled for their component MS spectrum. The two peaks at 12.06 sec and 12.46 sec match the mass of the H_4ABTC ligand. The two peaks present here correspond to the cis and trans form of H_4ABTC, as azobenzenes are known to undergo isomerization in the presence of UV light, with this isomerization changing the properties of the molecule.[7] A third peak corresponding to the mass of the thermally decarboxylated ligand was not detected in this sample.

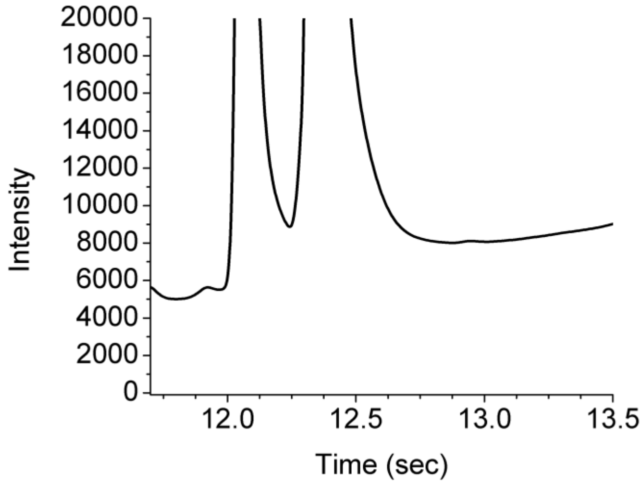

Figure S65. UV-GC Chromatogram of decomposed sample of PCN-250 (Al_3). The MOF was decomposed in pH=12 to yield the free H_4ABTC ligands in solution. The UV-GC Chromatagram was collected yielding three peaks, with each peak being sampled for their component MS spectrum. The two peaks at 12.21 sec and 12.48 sec match the mass of the H_4ABTC ligand. The two peaks present here correspond to the cis and trans form of H_4ABTC, as azobenzenes are known to undergo isomerization in the presence of UV light, with this isomerization changing the properties of the molecule.[7] A third peak corresponding to the mass of the thermally decarboxylated ligand was not detected in this sample.

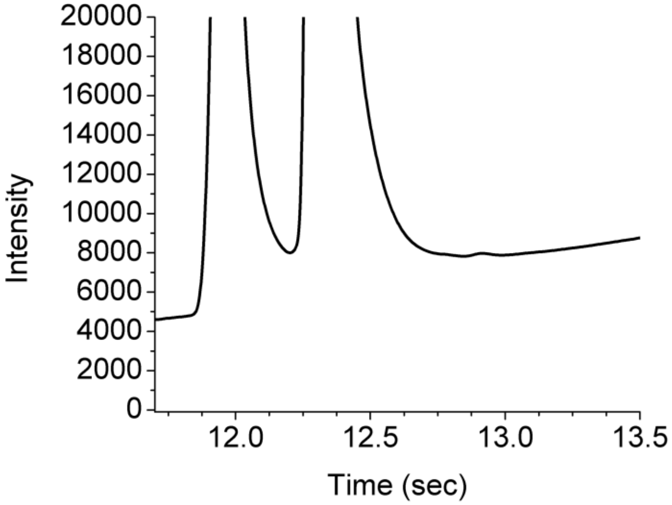

Figure S66. UV-GC Chromatogram of the decomposed sample of PCN-250 (In_3). The MOF was decomposed in pH=12 to yield the free H_4ABTC ligands in solution. The UV-GC Chromatagram was collected yielding three peaks, with each peak being sampled for their component MS spectrum. The two peaks at 12.18 sec and 12.54 sec match the mass of the H_4ABTC ligand. The two peaks present here correspond to the cis and trans form of H_4ABTC, as azobenzenes are known to undergo isomerization in the presence of UV light, with this isomerization changing the properties of the molecule.[7] A third peak corresponding to the mass of the thermally decarboxylated ligand was not detected in this sample.

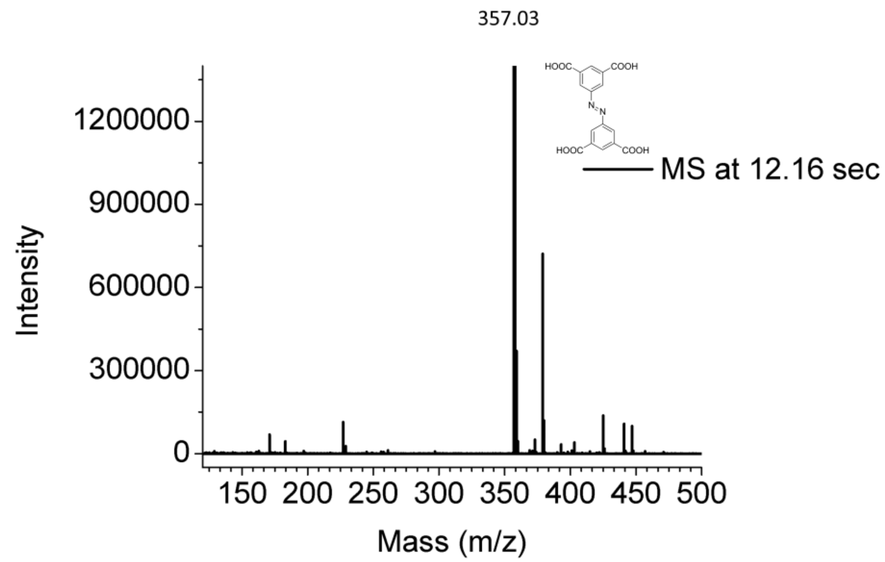

Figure S67. Sample Mass Spectrum of the GC-MS studies taken from the 12.16 sec peak which is present for all MOFs in the study. The major component of the spectrum corresponds to the mass of the H_4ABTC ligand.

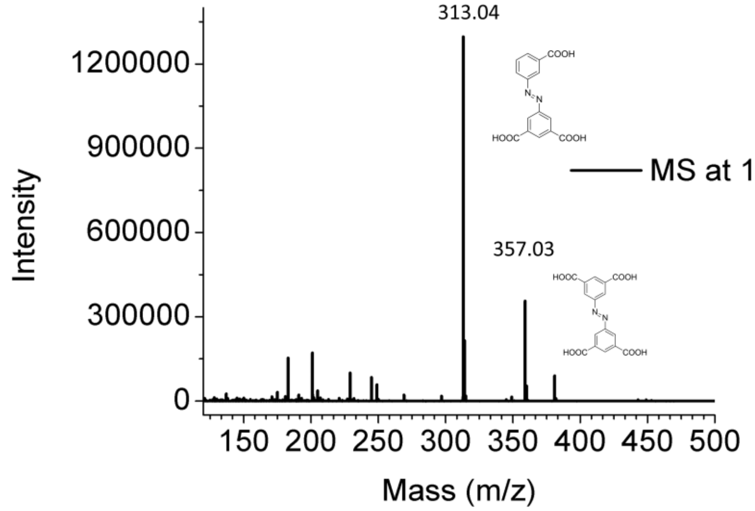

Figure S68. Sample Mass Spectrum of the GC-MS studies taken from the 13.06 sec peak that is present in the redox active cluster containing MOFs but not in the redox inert cluster containing MOFs. The major component of the spectrum corresponds to the mass of the decarboxylated H_4ABTC ligand. The H_4ABTC ligand is also present in the spectrum, but in a much smaller relative quantity.

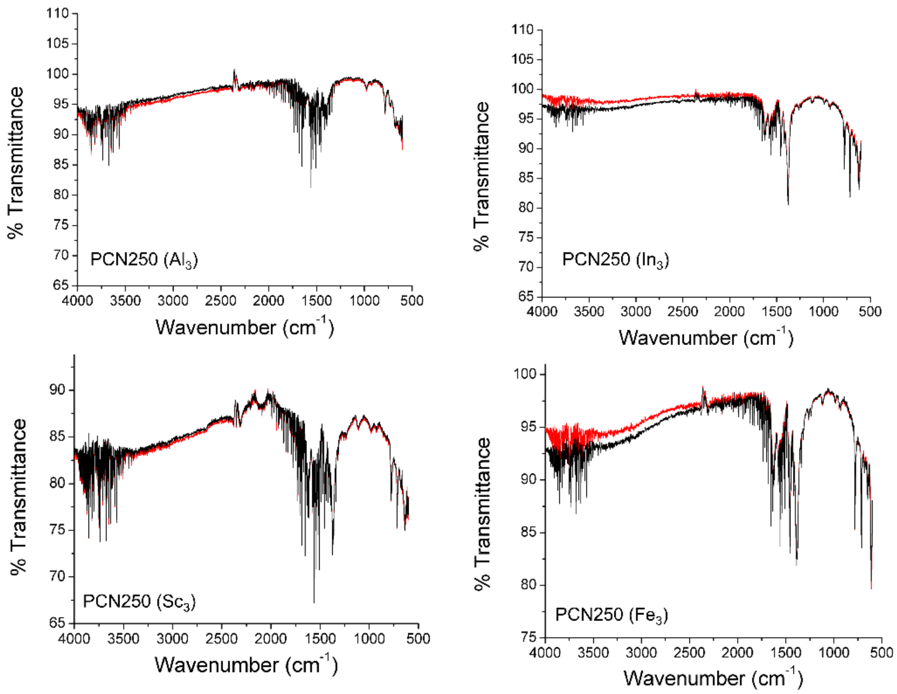

Figure S69. Infrared Spectroscopy of microporous freshly synthesized sample (red) and thermally treated samples (black) of PCN250 (Al_3), PCN250 (In_3), PCN250 (Sc_3), and PCN250 (Fe_3).

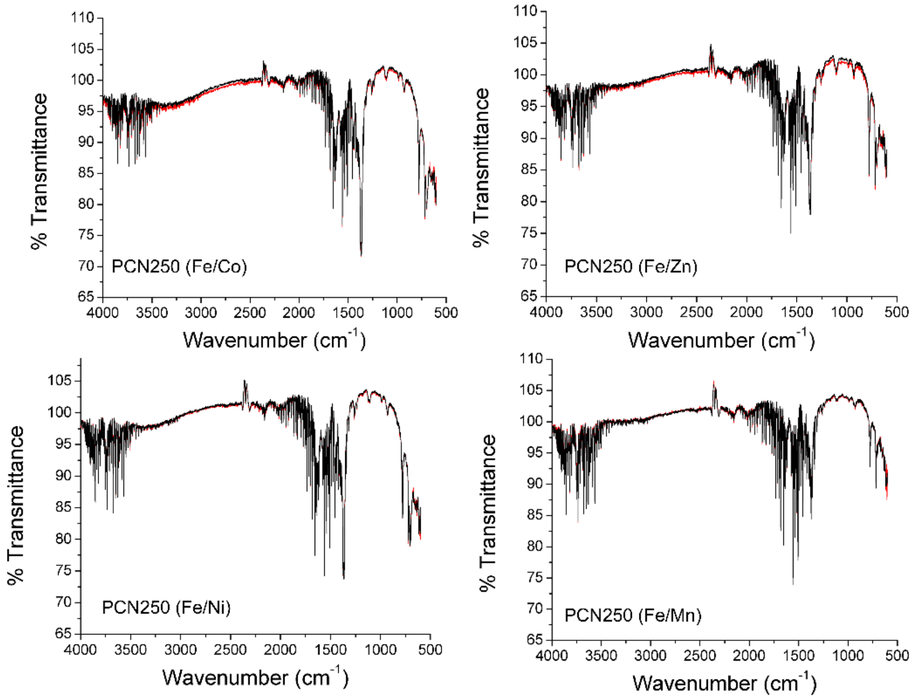

Figure S70. Infrared Spectroscopy of microporous freshly synthesized sample (red) and thermally treated samples (black) of PCN250 (Fe/Co), PCN250 (Fe/Zn), PCN250 (Fe/Ni), and PCN250 (Fe/Mn).

References

1. H. F. Drake, G. S. Day, S. W. Vali, Z. Xiao, S. Banerjee, J. Li, E. A. Joseph, J. E. Kuszynski, Z. T. Perry, A. Kirchon, O. K. Ozdemir, P. A. Lindahl and H.-C. Zhou, *Chem Commun*, 2019, **55**, 12769-12772.
2. K. Wang and H. Zhou, 2015.
3. Y. Fang, S. Banerjee, E. A. Joseph, G. S. Day, M. Bosch, J. Li, Q. Wang, H. Drake, O. K. Ozdemir, J. M. Ornstein, Y. Wang, T.-B. Lu and H.-C. Zhou, *Chem. - Eur. J.*, 2018, **24**, 16977-16982.
4. A. Dhakshinamoorthy, M. Alvaro, H. Chevreau, P. Horcajada, T. Devic, C. Serre and H. Garcia, *Catal. Sci. Technol.*, 2012, **2**, 324-330.
5. A. Kirchon, P. Zhang, J. Li, E. A. Joseph, W. Chen and H.-C. Zhou, *ACS Applied Materials & Interfaces*, 2020, **12**, 9292-9299.
6. M. Thommes, K. Kaneko, A. V. Neimark, J. P. Olivier, F. Rodriguez-Reinoso, J. Rouquerol and K. S. W. Sing, *Pure Appl. Chem.*, 2015, **87**, 1051-1069.
7. G. S. Kumar and D. C. Neckers, *Chem. Rev.*, 1989, **89**, 1915-1925.
8. I. Langmuir, *Journal of the American Chemical Society*, 1918, **40**, 1361-1403.
9. M. Králik, *Chemical Papers*, 2014, **68**, 1625-1638.
10. H. Swenson and N. P. Stadie, *Langmuir*, 2019, **35**, 5409-5426.
11. J. A. Mason, K. Sumida, Z. R. Herm, R. Krishna and J. R. Long, *Energy & Environmental Science*, 2011, **4**, 3030-3040.
12. H. Li, K. Wang, D. Feng, Y. P. Chen, W. Verdegaal and H. C. Zhou, *ChemSusChem*, 2016, **9**, 2832-2840.
13. W. Lu, W. M. Verdegaal, J. Yu, P. B. Balbuena, H.-K. Jeong and H.-C. Zhou, *Energy & Environmental Science*, 2013, **6**, 3559-3564.
